# Restoring Tumour Selectivity of the Bioreductive Prodrug PR-104 by Developing an Analogue Resistant to Aerobic Metabolism by Human Aldo-Keto Reductase 1C3

**DOI:** 10.3390/ph14121231

**Published:** 2021-11-26

**Authors:** Maria R. Abbattista, Amir Ashoorzadeh, Christopher P. Guise, Alexandra M. Mowday, Rituparna Mittra, Shevan Silva, Kevin O. Hicks, Matthew R. Bull, Victoria Jackson-Patel, Xiaojing Lin, Gareth A. Prosser, Neil K. Lambie, Gabi U. Dachs, David F. Ackerley, Jeff B. Smaill, Adam V. Patterson

**Affiliations:** 1 Auckland Cancer Society Research Centre, School of Medical Sciences, The University of Auckland, Auckland 1142, New Zealand; m.abbattista60@gmail.com (M.R.A.); a.ashoorzadeh@auckland.ac.nz (A.A.); chrispguise@hotmail.com (C.P.G.); a.mowday@auckland.ac.nz (A.M.M.); r.mittra038@gmail.com (R.M.); shevansilva116@gmail.com (S.S.); k.hicks@auckland.ac.nz (K.O.H.); m.bull@auckland.ac.nz (M.R.B.); v.jackson@auckland.ac.nz (V.J.-P.); x.lin@auckland.ac.nz (X.L.); j.smaill@auckland.ac.nz (J.B.S.); 2 Maurice Wilkins Centre for Molecular Biodiscovery, Auckland 1142, New Zealand; gabi.dachs@otago.ac.nz (G.U.D.); david.ackerley@vuw.ac.nz (D.F.A.); 3 School of Biological Sciences, Victoria University of Wellington, Wellington 6011, New Zealand; GProsser001@dundee.ac.uk; 4 School of Life Sciences, University of Dundee, Dundee DD1 5EH, UK; 5 Department of Anatomical Pathology, Canterbury Health Laboratories, Christchurch 8140, New Zealand; neil.lambie@cdhb.health.nz; 6 Department of Pathology and Biomedical Science, University of Otago, Christchurch 8140, New Zealand

**Keywords:** hypoxia-activated prodrug, bioreductive prodrug, PR-104, myelotoxicity, aldo-keto reductase 1C3, orthologues, cytochrome P450 oxidoreductase

## Abstract

PR-104 is a phosphate ester pre-prodrug that is converted in vivo to its cognate alcohol, PR-104A, a latent alkylator which forms potent cytotoxins upon bioreduction. Hypoxia selectivity results from one-electron nitro reduction of PR-104A, in which cytochrome P450 oxidoreductase (POR) plays an important role. However, PR-104A also undergoes ‘off-target’ two-electron reduction by human aldo-keto reductase 1C3 (AKR1C3), resulting in activation in oxygenated tissues. AKR1C3 expression in human myeloid progenitor cells probably accounts for the dose-limiting myelotoxicity of PR-104 documented in clinical trials, resulting in human PR-104A plasma exposure levels 3.4- to 9.6-fold lower than can be achieved in murine models. Structure-based design to eliminate AKR1C3 activation thus represents a strategy for restoring the therapeutic window of this class of agent in humans. Here, we identified SN29176, a PR-104A analogue resistant to human AKR1C3 activation. SN29176 retains hypoxia selectivity in vitro with aerobic/hypoxic IC_50_ ratios of 9 to 145, remains a substrate for POR and triggers γH2AX induction and cell cycle arrest in a comparable manner to PR-104A. SN35141, the soluble phosphate pre-prodrug of SN29176, exhibited superior hypoxic tumour log cell kill (>4.0) to PR-104 (2.5–3.7) in vivo at doses predicted to be achievable in humans. Orthologues of human AKR1C3 from mouse, rat and dog were incapable of reducing PR-104A, thus identifying an underlying cause for the discrepancy in PR-104 tolerance in pre-clinical models versus humans. In contrast, the macaque AKR1C3 gene orthologue was able to metabolise PR-104A, indicating that this species may be suitable for evaluating the toxicokinetics of PR-104 analogues for clinical development. We confirmed that SN29176 was not a substrate for AKR1C3 orthologues across all four pre-clinical species, demonstrating that this prodrug analogue class is suitable for further development. Based on these findings, a prodrug candidate was subsequently identified for clinical trials.

## 1. Introduction

Prodrugs have been developed that exploit oxygen depletion (hypoxia) in human solid tumours, a pervasive feature associated with resistance to radiotherapy [1], chemotherapy [2] and immunotherapy [3,4]. Cumulative evidence has firmly established the correlation between hypoxia and poor prognosis [5]. Examples of hypoxia-selective prodrugs that have been evaluated clinically include tirapazamine [6], porfiromycin [7], apaziquone [8], banoxantrone [9], evofosfamide [10], tarloxotinib [11] and PR-104 [12]. 

PR-104 is a water-soluble phosphate pre-prodrug which undergoes systemic conversion to the di-nitro-benzamide mustard (DNBM) alcohol prodrug PR-104A in vivo [12]. PR-104A then undergoes enzymatic reduction within cells. One-electron reduction of PR-104A generates a nitro radical anion intermediate. Hypoxia selectivity results from rapid back oxidation of this intermediate in the presence of molecular oxygen, whilst under hypoxic conditions further reduction leads to cytotoxic metabolite formation [12]. The dominant enzyme involved in one-electron reduction of PR-104A in vitro is cytochrome P450 oxidoreductase (POR) [13,14], but other related diflavin oxidoreductases are also capable of reducing PR-104A [15]. In addition, PR-104A was identified as a substrate for aldo-keto reductase 1C3 (AKR1C3) [16], a ketosteroid reductase which plays a key role in steroid hormone and prostaglandin regulation [17,18,19]. We discovered that AKR1C3 catalyses the concerted two-electron nitro reduction of PR-104A, a metabolic process that bypasses the nitro radical intermediate and is thus refractory to inhibition by molecular oxygen [16]. Metabolic reduction by AKR1C3 thus represents an off-target mechanism of bioactivation resulting in cytotoxin formation in a range of well-oxygenated normal tissues, including bone marrow progenitors. Reduction by either hypoxic or aerobic mechanisms leads to the formation of the cytotoxic hydroxylamine (PR-104H) and amine (PR-104M) metabolites [12,16].

In phase I clinical trials in patients with solid tumours, the maximum tolerated dose (MTD) of PR-104 was determined to be 1100 mg/m^2^ when administered once every 21 days (q3w) [20] or 675 mg/m^2^ when administered on days 1, 8 and 15 every 28 days (q1w) [21]. Dose-limiting toxicities (DLTs) were reported to be neutropenia, thrombocytopenia and infection with normal absolute neutrophil count. For patients treated using the q1w schedule, persisting thrombocytopenia limited treatment beyond two cycles in patients who received >270 mg/m^2^ [21]. A phase Ib study combining gemcitabine or docetaxel with PR-104 reported DLTs of thrombocytopenia or neutropenia at PR-104 doses >200 mg/m^2^; however, addition of prophylactic granulocyte colony stimulating factor (G-CSF) permitted PR-104 dose escalation to 770 mg/m^2^ in combination with docetaxel [22]. In hepatocellular carcinoma, the combination of sorafenib and PR-104 was discontinued due to thrombocytopenia and febrile neutropenia at doses of 555 or 770 mg/m^2^ [23].

**Figure 1:**
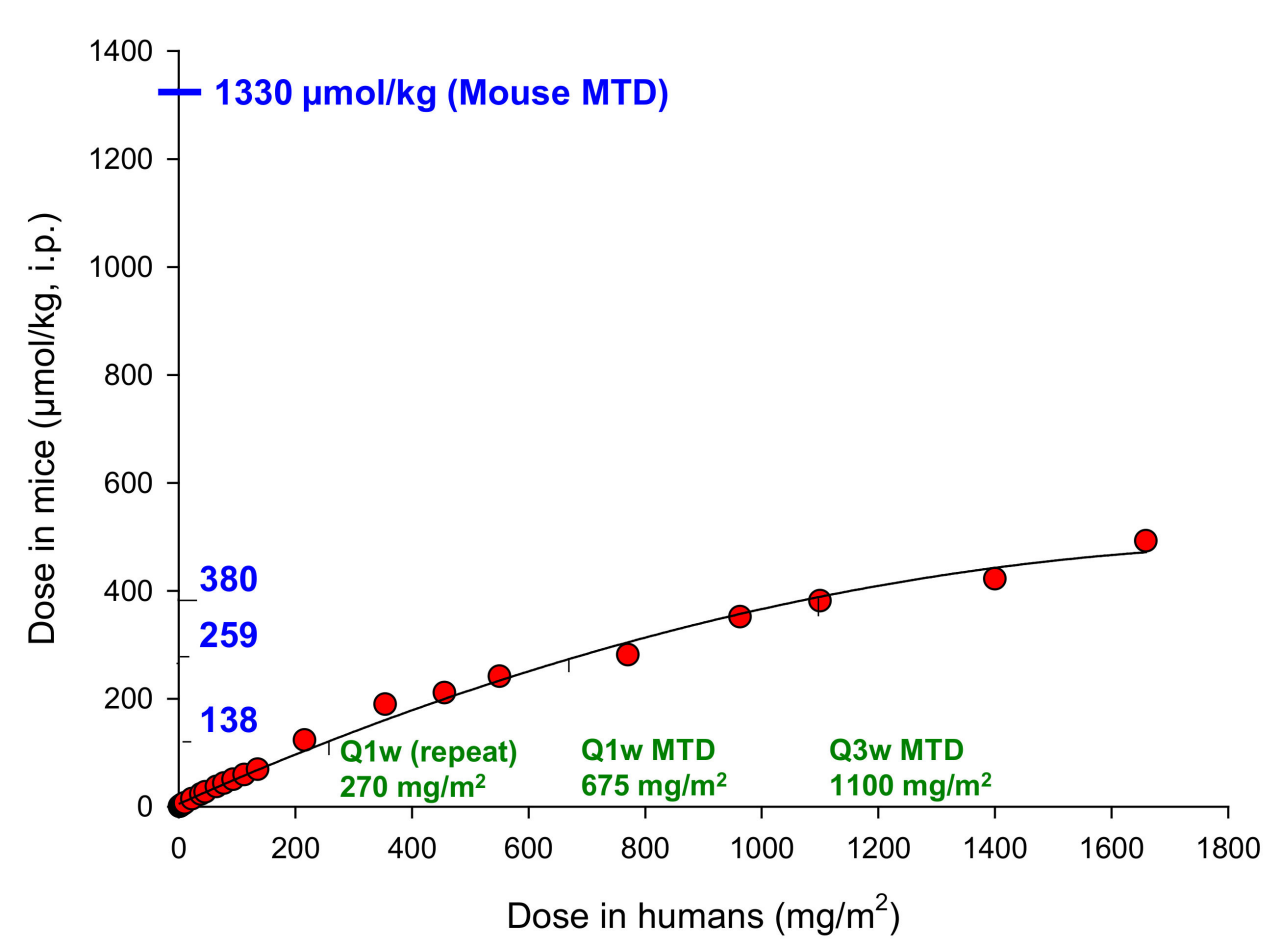
The relationship between the PR-104 input dose in mice and humans to achieve identical plasma exposure (AUC_inf_) of the prodrug PR-104A. Clinically relevant doses of PR-104 are indicated on the x-axis with the corresponding human equivalent dose (HED) in mice on the y-axis. The maximum safe dose of PR-104 in human subjects is 10% to 29% of that achieved in mice.

Overall, in a solid tumour setting, myelotoxicity prevents dose escalation of PR-104, restricting the area under the curve (AUC) of PR-104 in humans to levels below the point where pre-clinical activity is observed in human tumour xenograft models. The predicted plasma AUC of PR-104A was evaluated in humans, following intravenous infusion of PR-104 from 1.3 to 1660 mg/m^2^ [24]. The human equivalent doses of PR-104, corresponding to the q3w MTD (1100 mg/m^2^), the q1w MTD (675 mg/m^2^) and the q1w dose tolerated in repeat cycles (270 mg/m^2^), were calculated as 380, 259 and 138 µmol/kg (220, 150 and 80 mg/kg) in mice, respectively. This corresponds to 29%, 19% and 10% of the mouse MTD, based on the dose in mice that provides an equivalent plasma AUC_free_ to the human MTDs indicated [20,21,24,25] (Figure 1 and Table S1). This observed disconnect is associated with the severe myelotoxicity seen in human trials but not in mouse studies.

The clinical neutropenia and thrombocytopenia observed following administration of PR-104 indicates that human haematopoietic progenitor cells are susceptible to toxicity from PR-104A exposure. The likely mechanisms behind this toxicity include the expression of AKR1C3 in myeloid and erythroid cell lineages [26,27,28], the hypoxic environment within the bone marrow [29,30] or the presence of circulating cytotoxic metabolites in plasma [31]. Given the poor functional homology between human and murine AKR1C family members [32], we hypothesise that expression of AKR1C3 in myeloid progenitor cells is the primary mechanism underlying the dose-limiting toxicity of PR-104.

Here we report a novel analogue of PR-104A for which we have designed out metabolic activation by human AKR1C3. We confirm that SN29176 is resistant to human AKR1C3 metabolism, whilst hypoxia selectivity is retained. The mechanisms of cell cycle arrest and cell death are comparable to those observed for PR-104A [33] and remain dependent on the cellular complement of diflavin oxidoreductases. Further, the phosphate pre-prodrug of SN29176, termed SN35141, is refractory to AKR1C3 activation in vivo but retains promising efficacy in combination with radiotherapy in human tumour xenograft models. In order to identify the appropriate pre-clinical species for toxicology studies of novel analogues such as SN35141, we expressed commercially synthesised cDNAs from a series of AKR1C3 orthologues from various species (mouse, rat, dog, macaque and human) in HCT116 cells. Only cells expressing human and macaque AKR1C3 cDNA were sensitive to PR-104A (but not SN29176), reflecting the high sequence homology of the AKR1C family members between human and monkey [34].

## 2. Results

### 2.1. Human Haematopoietic Cells Are More Sensitive to PR-104A Than Murine Haematopoietic Cells

**Figure 2:**
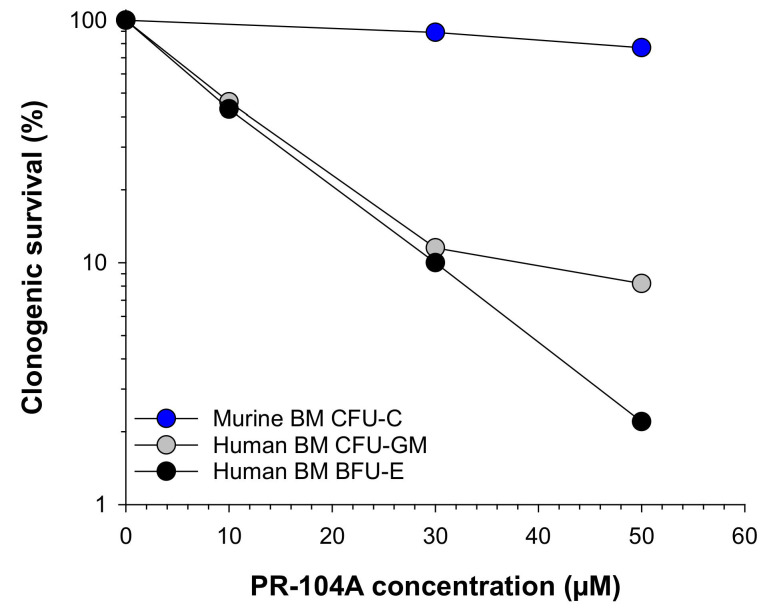
The acute myelotoxicity of PR-104 in human clinical studies is probably associated with the observed hypersensitivity of bone marrow progenitor cells to PR-104A exposure under normoxic conditions (21% O_2_). There is a large disparity in clonogenic survival of mouse and human bone marrow progenitor cells after aerobic exposure to PR-104A. Blue solid symbol (●): murine bone marrow colony forming units (cell) (MBM CFU-C); Grey solid symbol (●): human bone marrow colony forming units (granulocytes/macrophages) (HBM CFU-GM); Black solid symbol (●): human bone marrow burst forming units: (erythroid) (HBM BFU-E).

To determine whether expression of AKR1C3 in myeloid progenitor cells is a possible mechanism of the dose-limiting toxicity observed in humans, we first compared the aerobic sensitivity of murine and human bone marrow cells to PR-104A exposure under normoxia (21% O_2_). Human granulocyte/macrophage and erythroid progenitor cell populations were hypersensitive to aerobic PR-104A exposure, whereas murine haematopoietic progenitor cells were refractory (Figure 2). The observation that human haematopoietic cells are hypersensitive to the *aerobic* toxicity of PR-104A indicates the likely cause of haematological toxicity encountered by PR-104-treated patients. This is consistent with the reports of high expression of AKR1C3 in CD34^+^-positive human haematopoietic progenitor cells [26] and the lack of functional homology between murine and human AKR1C3 enzymes [32]. In toxicology studies, no evidence of myelotoxicity is observed in mice following PR-104 administration, with gastrointestinal toxicity identified by histology as a DLT above 1330 μmol/kg (770 mg/kg, not shown). Aerobic nitro reduction of PR-104A to its cytotoxic species is specific to human AKR1C3 [16], thereby supporting the interpretation that AKR1C3 catalytic activity is the major determinant of the discrepancy between the achievable PR-104 AUCs in pre-clinical mouse models and in clinical trials in humans (Figure 1).

### 2.2. Design of an AKR1C3-Resistant Nitrobenzamide Mustard

**Figure 3:**
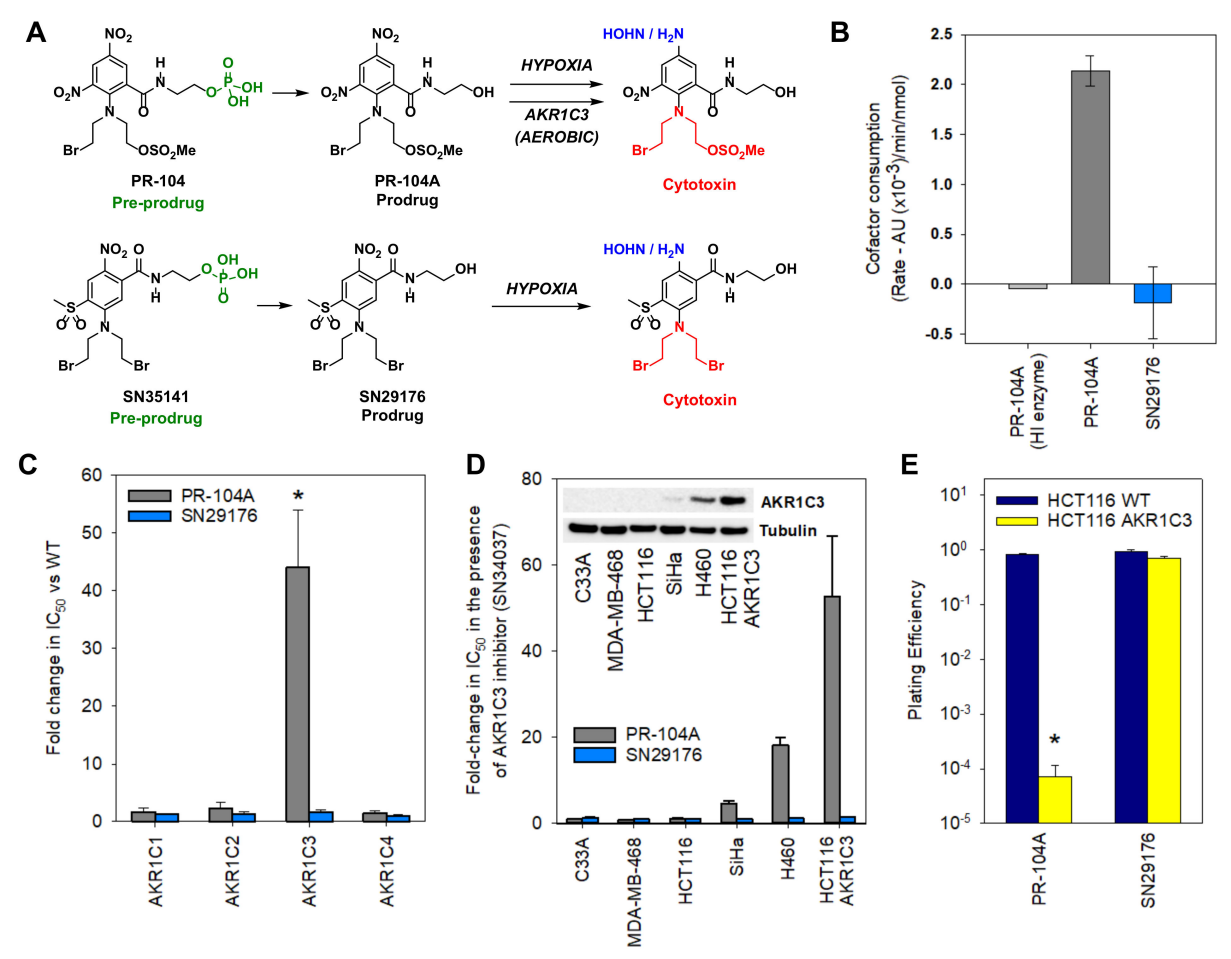
SN29176 is a novel PR-104A analogue that is resistant to aerobic bioactivation by human aldo-keto reductase 1C3 (AKR1C3). (**A**) Schematic of phosphate pre-prodrug PR-104 or SN35141 conversion to prodrug PR-104A and SN29176, respectively, with subsequent reduction to cytotoxic hydroxylamine and amine metabolites. (**B**) Recombinant human AKR1C3-enzyme-dependent consumption of co-factor NADPH upon incubation with PR-104A or SN29176. HI = heat inactivated. (**C**) Relative sensitivity of HCT116 cell lines expressing the AKR1C1-4 family members determined as the concentration of PR-104A or SN29176 required to inhibit cell growth by 50% compared to untreated controls following 4 h drug exposure and five days’ regrowth. Fold change in IC_50_ values indicates the ratio of the IC_50_ values between the untransfected (wild-type, WT) and AKR1C-expressing cell lines. (**D**) Relative sensitivity of cancer cell lines to aerobic exposure to PR-104A or SN29176 in the presence or absence of the potent, selective AKR1C3 inhibitor SN34037. Fold change in IC_50_ values indicates the ratio of the IC_50_ values in the presence and absence of inhibitor. Expression levels of AKR1C3 in the cell lines is determined by Western blotting (inset). (**E**) Average plating efficiency of HCT116 WT and HCT116 AKR1C3 cell lines following exposure to PR-104A or SN29176 (10 µM) at high cell density; * *p* < 0.001.

**Scheme 1:**
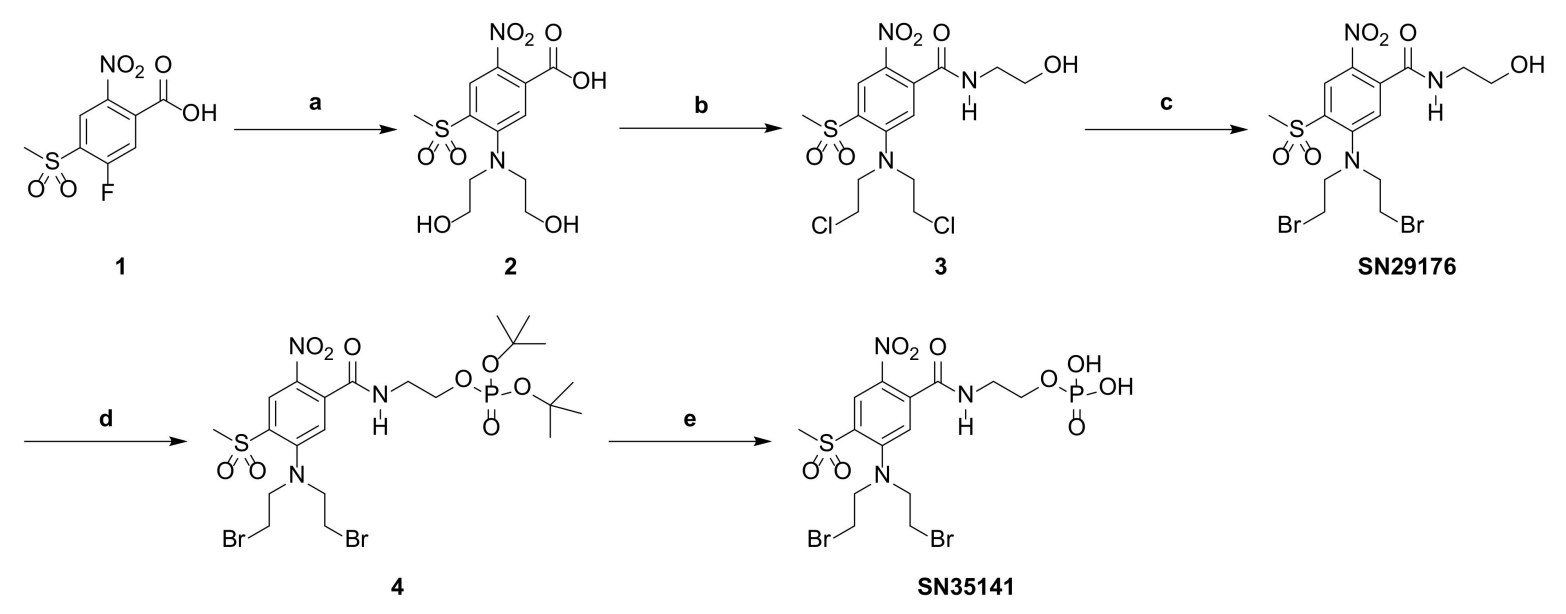
Synthesis of SN29176 and SN35141. Reagents and conditions: (**a**) diethanolamine, DMSO, R.T., 2 h; (**b**) (i) SOCl_2_, cat. DMF, reflux, 4 h, (ii) 2-aminoethanol, CH_2_Cl_2_, THF, 0 °C to R.T., 20 min; (**c**) LiBr, 3-methyl-2-butanone, reflux, 12 h (3×); (**d**) (i) di-*tert*-butyl-*N*,*N*-diisopropylphosphoramidite, 1*H*-tetrazole (3% in CH_3_CN), DMF, 5 °C to R.T., 4 h; (ii) *m*-CPBA (70%), CH_2_Cl_2_, 0 °C to R.T., 1 h; (**e**) TFA, CH_2_Cl_2_, 5 °C to R.T., 1 h.

Previous examination of dinitro benzamide mustard (DNBM) regio-isomers showed that hypoxia selectivity was evident in three isomer classes: the 2,4-DNBM, the 3,5-DNBM and the 2,6-DNBM [35,36] classes. In recombinant AKR1C3 assay screens, the 3,5-DNBM class (exemplified by PR-104A) exhibited high rates of aerobic metabolism by human AKR1C3 (Supplementary Figure S1), consistent with published findings [16]. In pre-clinical studies, the 2,6-DNBM isomer class displayed an unfavourable toxicology profile and was not considered for further development (data not shown). The remaining isomers, the 2,4-DNBMs, displayed appropriate hypoxia selectivity but are predominantly reduced at the 4-nitro group *ortho* to the mustard, and the resulting 4-amine/4-hydroxylamine reduction products undergo spontaneous molecular cyclisation to a weakly cytotoxic mono-mustard product [37,38]. Therefore, synthetic chemistry methods were developed (Scheme 1) to allow substitution of the 4-nitro with a group with similar electron-withdrawing potential, namely, a 4-methylsulfone, ensuring that reduction proceeds exclusively at the 2-nitro position (Figure 3A).

### 2.3. Chemistry

The synthesis of SN29176 from nitrobenzoic acid 1 [39,40] via six synthetic steps has been described by our laboratory in the patent literature [41]. In the present work, a more direct three-step procedure was developed that has also been published in patents [42]. Briefly, diethanolamine displacement of the activated 4-fluoro substituent of 1 provided carboxylic acid 2 (Scheme 1). Thionyl-chloride-mediated trichlorination and subsequent amide coupling of the resultant acid chloride intermediate with 2-aminoethanol then gave the dichloro mustard 3. Chlorine to bromine halogen exchange employing repeat submissions to lithium bromide in 3-methyl-2-butanone at reflux gave SN29176. A further two synthetic steps provided SN35141. Specifically, the 1*H*-tetrazole-mediated reaction with di-*tert*-butyl-di-*iso*-propylphosphoramidite followed by in situ oxidation with *meta*-chloroperoxybenzoic acid gave the di-*tert*-butyl phosphate ester 4, which was hydrolysed under acidic conditions to afford the phosphate pre-prodrug SN35141.

### 2.4. SN29176 Is Not a Substrate for Human AKR1C Enzymes

The ability of recombinant AKR1C3 to metabolise prodrugs was determined by measuring the loss of co-factor (NADPH) upon incubation of the prodrug with AKR1C3. Steady co-factor consumption was observed for PR-104A, whilst heat inactivation of AKR1C3 prevented this process. In contrast, no loss of co-factor was observed for SN29176 (Figure 3B). We next evaluated SN29176 in isogenic HCT116 cell lines overexpressing the human AKR1C subfamily (1C1, 1C2, 1C3 and 1C4) of enzymes [16]. In contrast to PR-104A, for which AKR1C3-expressing cells showed a 44-fold increase in aerobic sensitivity (*p* < 0.001), no changes in sensitivity to SN29176 were observed (Figure 3C). Anti-proliferative IC_50_ assays were also conducted using a panel of cancer cell lines with varying levels of endogenous AKR1C3 expression in the presence or absence of the AKR1C3 selective inhibitor SN34037 [43,44] (Figure 3D). Inhibition of AKR1C3 by SN34037 did not alter the sensitivity to SN29176, while it prevented sensitivity to PR-104A in a manner directly proportional to AKR1C3 expression (Figure 3D and Supplementary Figure S2a). We next evaluated the role of AKR1C3 in high-cell-density multi-cellular layers (MCLs) consisting of 100% HCT116 wild-type (WT) or 100% HCT116 AKR1C3 cells (Figure 3E). When treated with 10 µM PR-104A, plating efficiency in AKR1C3-expressing MCLs was >11,000-fold lower than in WT MCLs (*p* < 0.001). In contrast, AKR1C3 MCLs were no more sensitive to 10 µM SN29176 than WT MCLs.

### 2.5. SN29176 Shows Hypoxia Selectivity In Vitro and Is a Substrate for POR

**Figure 4:**
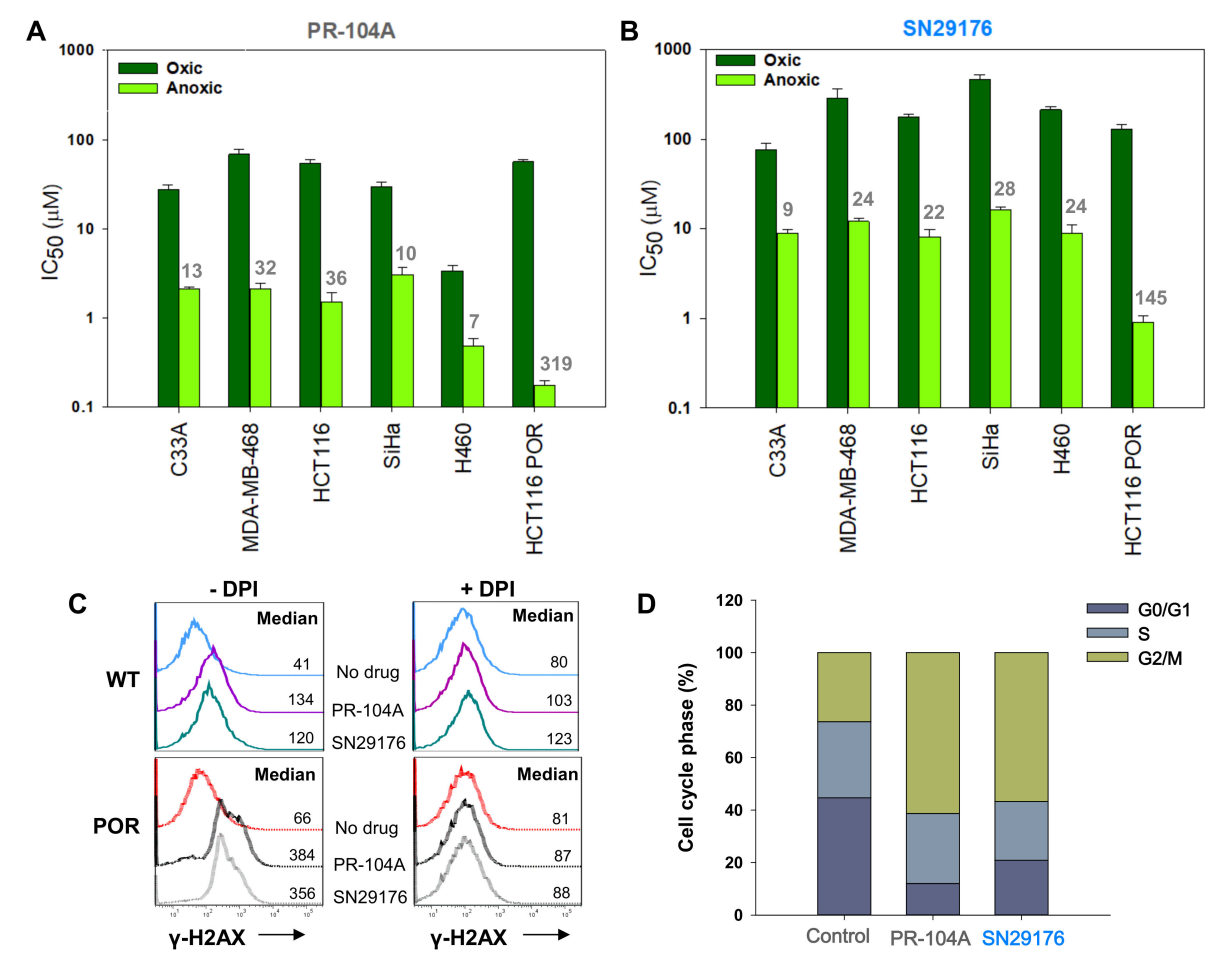
Conserved mechanism of action of SN29176 and PR-104A in vitro, with similar hypoxia selectivity and one-electron bioactivation to DNA-damaging species that induce cell cycle arrest. In vitro anti-proliferative activity of (**A**) PR-104A and (**B**) SN29176 in various cancer cell lines under aerobic and anoxic conditions. IC_50_ values were determined as the concentration of drug required to inhibit cell growth by 50% compared to untreated controls following 4 h drug exposure and five days’ regrowth (mean ± SD of three or more independent determinations). Numbers above the light green bars are the ratios of aerobic and anoxic conditions IC_50_ values (HCR = hypoxic cytotoxicity ratio). (**C**) Monitoring of DNA damage response in HCT116 WT and POR-expressing cells by immunocytochemical detection of γ-H2AX induction following PR-104A and SN29176 treatment under anoxia, with inhibition by diphenyleneiodonium (DPI), a suicide inhibitor of POR. (**D**) Change in cell cycle distribution in C33A cells following exposure to PR-104A or SN29176 at their respective IC_50_ values.

Hypoxia cytotoxicity ratios (HCRs; aerobic IC_50_ values divided by anoxic IC_50_ values) for AKR1C3-negative and AKR1C3-positive cell lines were evaluated (Figure 4A,B). In the AKR1C3-positive cell lines SiHa and H460, HCRs for PR-104A were suppressed relative to SN29176 (10 vs. 28 and 7 vs. 24, respectively) whereas values were broadly similar across the AKR1C3-negative cell lines. Importantly, POR overexpression increases the hypoxia selectivity (HCR) of SN29176 (22 vs. 145) in HCT116 cells; a phenomenon also seen for PR-104A (36 vs. 319) in this AKR1C3-negative cellular background (Figure 4A,B).

To compare the extent of DNA damage induced by SN29176 and PR-104A, we examined the phosphorylation of the DNA histone variant H2AX (γH2AX) in HCT116 WT and POR-expressing cells by flow cytometry, using prodrug concentrations corresponding to anoxic HCT116 IC_50_ values. Under anoxia, both PR-104A and SN29176 induced γH2AX, with greater intensity in HCT116 POR cells (Figure 4C), which were readily inhibited by pre-incubation with the flavoenzyme inhibitor diphenyliodonium (DPI) (Figure 4C). The induction of γH2AX DNA foci following anoxic exposure of HCT116 POR cells was associated with loss of clonogenic potential and was consistently reversed by DPI (Supplementary Figure S2b). In contrast, no γH2AX induction was observed under aerobic conditions irrespective of POR expression or DPI pre-exposure, confirming the strict dependence on one-electron reduction by POR to elicit a DNA damage response (Supplementary Figure S3). To determine whether DNA damage induced by SN29176 or PR-104A under anoxia caused cell cycle arrest, C33A cells were stained with propidium iodide and analysed by flow cytometry (Figure 4D). At 24 h post treatment, both PR-104A and SN29176 arrested C33A cells in the G2/M phase, with the percentage of cells in the G2/M phase increasing from 26.32% (control) to 61.31% (post PR-104A treatment) and 56.76% (post SN29176 treatment). Collectively, these data indicate a shared mechanism of action for PR-104A and SN29176.

### 2.6. SN35141, the Phosphate Pre-Prodrug of SN29176, Is Active in Human Tumour Xenografts

**Figure 5:**
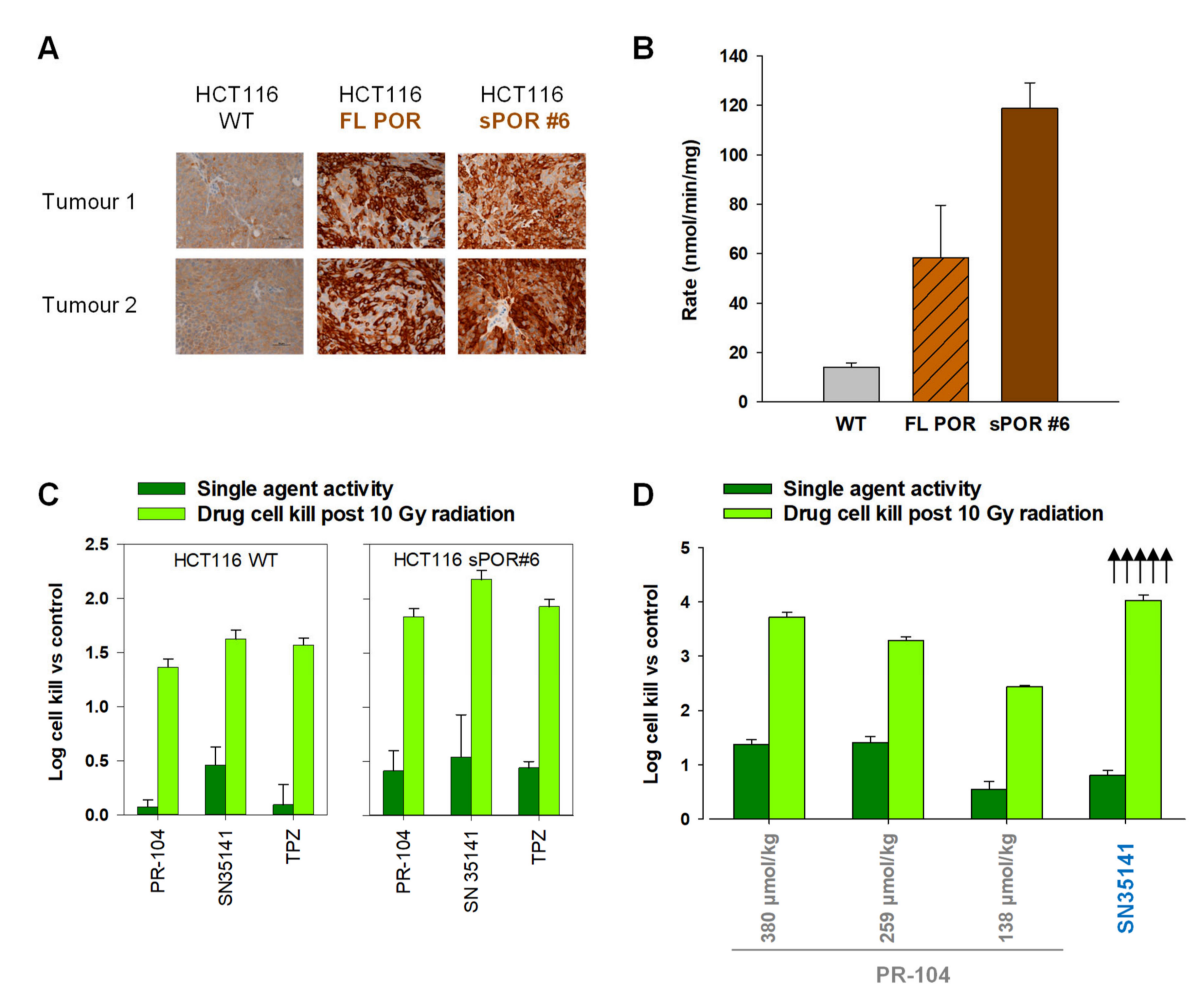
Evaluation of the therapeutic efficacy of SN35141, the water-soluble phosphate pre-prodrug of SN29176. (**A**) Detection of POR in xenografts of the clonal cell line sPOR#6 with representative immunohistochemistry. (**B**) NADPH-cytochrome C reduction assay on a clonal cell line of HCT116 generated to homogeneously overexpress a soluble version of POR in xenografts in which the endoplasmic reticulum membrane anchor has been deleted (sPOR#6). FL = full length POR. (**C**) Clonogenic cell kill of HCT116 WT and HCT116 sPOR#6 xenografts 18 h after treatment with hypoxia-activated prodrugs PR-104 (380 µmol/kg, i.p.), SN35141 (1330 µmol/kg, i.p.) or tirapazamine (178 µmol/kg, i.p.). (**D**) Clonogenic cell kill of AKR1C3-positive SiHa xenografts after treatment with PR-104A at human equivalent doses (as indicated) and SN35141 at 75% MTD (1330 µmol/kg). Arrows indicate 5/10 tumours yielded zero viable colonies (<1 viable clonogen/300,000 cells).

To develop a mixed clonal cell line exhibiting strong POR expression for use in tumour xenograft models, we initially overexpressed full-length POR (FL POR), but this resulted in micro-heterogenous staining intensity in xenografts (Figure 5A). To address this problem, we removed the N-terminal ER localisation signal to generate a soluble, cytosolic POR variant (sPOR), thereby facilitating a more homogeneous expression (Figure 5A). Individual HCT116 sPOR clones were isolated, and those with high POR activity were identified by cytochrome C reduction assay (Supplementary Figure S4A). Seven clones were evaluated in vivo, three of which (#2, #6 and #12) showed homogenous sPOR expression and high ex vivo POR activity (Supplementary Figure S4B,C). Of the three clones, #6 exhibited the greatest ex vivo plating efficiency (Figure S5A), strong and homogeneous POR expression when compared to heterogeneous expression in FL POR xenografts (Figure 5A and Supplementary Figure S5B) and high levels of POR activity (Figure 5B). Ex vivo populations of HCT116 sPOR#6 were therefore used for subsequent in vivo experiments.

Initially, the maximum tolerated dose (MTD) of SN35141 was defined in nude mice. A single dose of 1770 µmol/kg (ip) was well tolerated with no evidence of morbidity or mortality accompanied by a minimal body weight loss (BWL) nadir of −2.7% ± 1.9%, *n* = 7 mice (MTD defined as <−15% average BWL). Next, we compared the efficacy of SN35141 (1330 µmol/kg) against PR-104 (380 µmol/kg, HED) or TPZ (178 µmol/kg, MTD) in combination with ionising radiation (10 Gy) in HCT116 xenografts (Figure 5C). SN35141 showed comparable activity to PR-104 or TPZ in this model. The observed cell kill post radiation (of the radiation-resistant anoxic cell population) in HCT116 WT xenografts was in the range of 1.36–1.62 log cell kill for all prodrugs. However, increases in post-radiation log cell kill were observed in the isogenic xenograft model overexpressing POR, ranging from 1.84–2.18 log cell kill. Log cell kill was greatest for SN35141 in both models at the doses tested. We then evaluated the activity of SN35141 versus PR-104 in SiHa xenografts (Figure 5D). PR-104 was dosed at 380, 259 and 138 µmol/kg (corresponding to HEDs of 1100 mg/m^2^, 675 mg/m^2^ and 270 mg/m^2^, respectively), whilst SN35141 was dosed at 75% ofMTD (1330 µmol/kg). The greatest single agent activity was observed for PR-104, probably due to aerobic activation of PR-104A by AKR1C3 in SiHa cells (Figure 3D). However, when the prodrug was administered in combination with 10 Gy radiation, SN35141 exhibited superior hypoxic cell kill compared to PR-104, with 5/10 tumours showing cell kill beyond the limit of detection (<1 viable clonogen per 300,000 cells).

### 2.7. SN35141 Is Not a Substrate for AKR1C3

**Figure 6:**
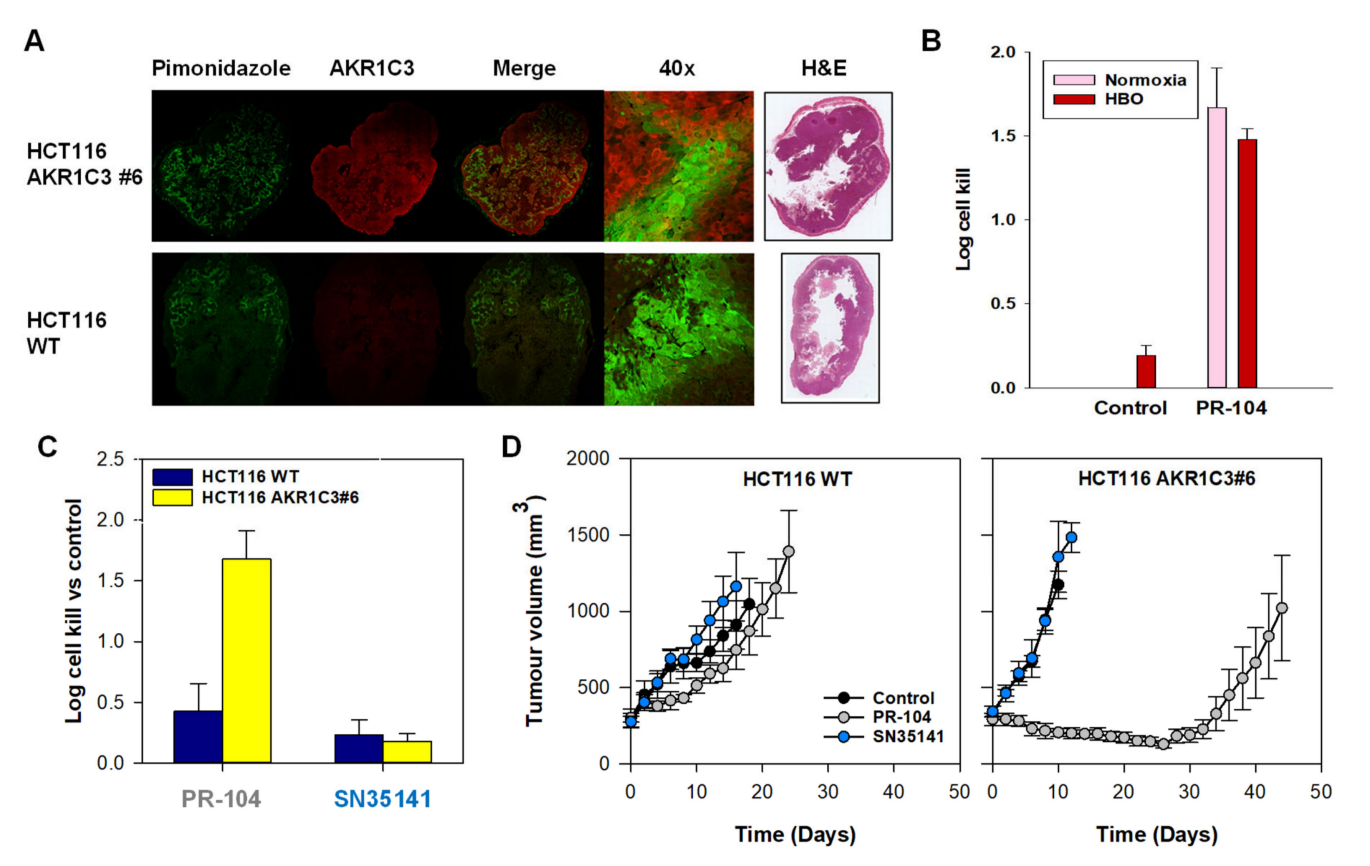
SN35141/SN29176 is not a substrate for AKR1C3 in vivo. (**A**) Detection of hypoxia (via pimonidazole) and AKR1C3 in a clonal cell line of HCT116 generated to homogeneously overexpress AKR1C3 in xenografts (AKR1C3#6) with representative immunofluorescence. (**B**) Clonogenic cell kill of PR-104-treated (380 µmol/kg) HCT116 AKR1C3#6 xenografts +/− hyperbaric oxygen (HBO, 100% O_2_, 32 psi, 2 h). Log cell kill values are relative to normoxic controls. (**C**) Clonogenic cell kill of HCT116 WT and HCT116 AKR1C3#6 xenografts after treatment with PR-104 at the human equivalent dose (380 µmol/kg) or SN35141 at 75% MTD (1330 µmol/kg). (**D**) Inhibition of growth of HCT116 WT or HCT116 AKR1C3#6 xenografts by PR-104 or SN35141 monotherapy. Both prodrugs were administered as a single dose of 1330 µmol/kg on day 0.

As for POR, we initially sought to develop a clonal cell line exhibiting strong AKR1C3 expression for in vivo studies. The AKR1C probe coumberone was used to determine enzyme expression in HCT116 AKR1C3 clones in vitro (Supplementary Figure S6A). Clones were then implanted into mice and xenografts (2–3 mice per clone) were excised when they were 9–10 mm in diameter. Single-cell suspensions of tumours were analysed for coumberone metabolism (Supplementary Figure S6B) and plating efficiency (Supplementary Figure S6C). AKR1C3 expression was also evaluated by immunohistochemistry (Supplementary Figure S6D). All clones showed increased rates of coumberone metabolism compared to WT and strong homogenous expression of AKR1C3. HCT116 AKR1C3#6 showed the least variability across assays and was selected as the preferred clone, and ex vivo populations of HCT116 AKR1C3#6 were used in subsequent in vivo experiments. The relative metabolic capacities of the sPOR#6 and AKR1C3#6 tumour models were established in vivo by monitoring PR-104A reduction in vivo (Supplementary Figure S7). After confirming that overexpression of AKR1C3 in HCT116 AKR1C3#6 did not modify the hypoxic fraction (Figure 6A), we used the new HCT116 AKR1C3#6 clone to investigate the relationship between PR-104 activity and tumour oxygenation status. Exposure of HCT116 AKR1C3#6 tumour-bearing mice to hyperbaric oxygen (100% O_2_, 32 psi) did not reduce the therapeutic efficacy of PR-104 (Figure 6B), confirming that AKR1C3 expression plays a dominant role.

Next, we utilised HCT116 WT and HCT116 AKR1C3#6 xenograft models to test the comparative role of AKR1C3 in the efficacy of PR-104 and SN35141 in vivo. SN35141 was administered at 75% of maximum tolerated dose (MTD, 1330 µmol/kg) and PR-104 at a human equivalent dose (HED, 380 µmol/kg) in an excision assay with a clonogenic end point (Figure 6C). Both prodrugs produced modest levels of cell kill in (AKR1C3-negative) HCT116 WT xenografts; PR-104 and SN35141 caused modest cell kill relative to drug-free controls (0.43 and 0.23 logs, respectively). Expression of AKR1C3 significantly enhanced the level of cell kill due to PR-104 (0.43 versus 1.68 log cell kill, *p* < 0.01) but not SN35141 (0.23 versus 0.18 log cell kill, ns). The influence of AKR1C3 on prodrug efficacy was also assessed by tumour growth delay (Figure 6D). Expression of AKR1C3 resulted in significant tumour control following a single dose of PR-104 (1330 µmol/kg) but not SN35141 (1330 µmol/kg), thereby confirming the resistance of SN35141 to this hypoxia-independent off-target activity.

### 2.8. The Macaque Monkey Is a Suitable Pre-Clinical Animal Model for Evaluation of SN35141

Isogenic HCT116 cell lines expressing mouse, rat, dog and macaque monkey AKR1C3 orthologues, as well as the macaque AKR1C1 and AKR1C4 orthologues, were generated (complete list of sequence sources in Table S1). 

**Figure 7:**
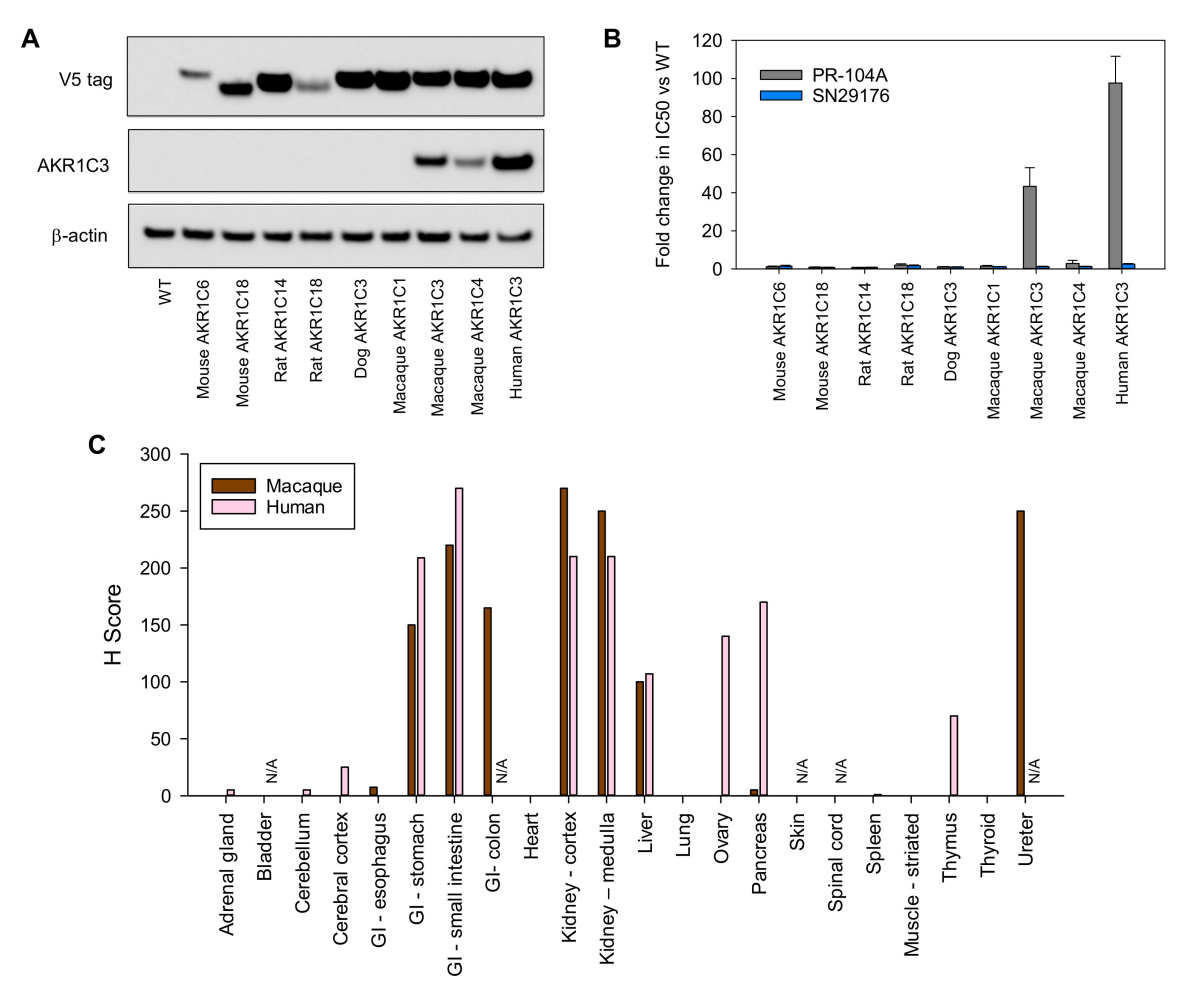
The macaque monkey AKR1C3 orthologue sensitises cells to PR-104A, indicating it is a suitable animal model for pre-clinical evaluation of SN29176. (**A**) Western blot confirming codon-optimised AKR1C3 orthologue expression in stably transfected HCT116 cells. (**B**) In vitro anti-proliferative activity with PR-104A and SN29176 in HCT116 cell lines expressing codon-optimised AKR1C3 orthologues. IC_50_ values were determined as the concentration of drug required to inhibit cell growth by 50% compared to untreated controls following 4 h drug exposure, with washing and regrowth for five days. Fold change in IC_50_ values indicates the ratio of the IC_50_ values between the untransfected (WT) and AKR1C3 orthologue cell lines. (**C**) Comparison of the AKR1C3 staining intensity (H-score) in normal human and macaque tissue. N/A = not assessed.

Protein expression was confirmed via an inducible V5 tag (Figure 7A). An antibody selective for AKR1C3 in humans was shown to cross react with macaque AKR1C3 and AKR1C4 (Figure 7A). The sensitivity of these cell lines to PR-104A and SN29176 was then evaluated. Mouse, rat and dog orthologues of AKR1C3 were inactive for both prodrug substrates (for sequence homologies see Supplementary Figure S8). Increases in sensitivity were only observed when cells expressing macaque or human AKR1C3 were exposed to PR-104A. As expected, no increases in sensitivity to SN29176 were observed (Figure 7B). Previously, we evaluated AKR1C3 expression by immunohistochemistry in microarrays consisting of sections of human tumour or normal tissues [16]. Here, we evaluated AKR1C3 expression in a microarray of 22 normal macaque tissue sections using the same high-quality anti-AKR1C3 monoclonal antibody (Figure 7C). Staining intensity and distribution (H-score) of AKR1C3 in macaque tissues was similar to that seen in human tissues with the exception of ovary, pancreas and thymus, which showed lower AKR1C3 expression than observed previously [16] in human tissues (Figure 7C).

## 3. Discussion

Scientists have long sought agents to eliminate hypoxia within the tumour microenvironment, particularly through the design of hypoxia-activated prodrugs (HAP), i.e., ‘masked’ agents that are bioactivated under O_2_-limiting conditions [45,46,47]. Despite the conceptual appeal and urgent need, clinical success with HAP remains elusive, benchmarked most visibly by the failure of tirapazamine and evofosfamide in phase 3 trials [48,49,50,51]. Given that over half of all human tumours harbour pathophysiological hypoxia (pO_2_ < 1%) [52], a successful HAP technology would deliver major clinical impact. PR-104 was intended to address this unmet need but encountered unexpected early challenges during clinical development. Specifically, the maximum safe exposure to PR-104 in patients with solid tumours was 3.4- to 9.6-fold lower than could be achieved in pre-clinical murine models, as defined by total plasma exposure (AUC_free_) to unbound prodrug PR-104A (Figure 1) [22,25]. This toxicokinetic disconnect is unusual for alkylating agents which typically scale in a predictable 1:1 fashion between murine and human subjects [53]. The hypersensitivity of human bone marrow progenitor cells to *aerobic* PR-104A exposure in vitro (Figure 2) indicates a strong causal link with the clinical myelotoxicity observations. PR-104A is designed to remain inert under normoxia; aerobic cytotoxicity thus discloses the presence of off-target metabolic activity. An important endogenous catalytic role of AKR1C3 is as a prostaglandin D_2_ 11-ketoreductase (prostaglandin F synthase) which regulates maturation of CD34+ myeloid progenitor cells [17,26]. AKR1C3 also acts as a unique aerobic nitro reductase that can bioactivate PR-104A under oxygenated conditions [16]. This overlapping functionality in early lineage bone marrow cells is thus thought to be a major source of the grade 3/4 myelotoxicity reported at low doses of PR-104 in clinical trials. 

Based on this evidence, we synthesised and characterised an analogue of PR-104 (SN35141, Scheme 1, Figure 3A) that is resistant to activation by AKR1C3, thereby reinstating the original design concept of selective activation under hypoxia. In vitro metabolism and 2D (low-density) cytotoxicity assays confirmed that SN29176 is not a substrate for two-electron reduction by human AKR1C3 (Figure 3B–E). The scale of this difference was most apparent when employing high-cell-density in vitro 3D MCL models (Figure 3E) and in vivo tumour models (Figure 6C,D), reflecting the tendency of the lipophilic metabolites to redistribute locally (bystander effect). Since it was confirmed that SN29176 is not a substrate for AKR1C3, murine tolerance to the pre-prodrug SN35141 should, in principle, better predict the exposures achievable in human subjects [53]. 

Critically, the hypoxia-selective properties of SN29176 remain intact with HCR values ranging from 9 to 145 (Figure 4B), indicating the 2-nitro, 4-methylsulfone design functions as intended. To confirm the relationship between one-electron reduction of the prodrug and resulting DNA damage, we compared the formation of γH2AX foci in prodrug-exposed WT and POR-expressing HCT116 cells under anoxia, with or without the flavoenzyme inhibitor DPI. Both PR-104A and SN29176 exposure amplified the DNA damage response in a POR-dependent manner, a phenomenon prevented by prior DPI exposure (Figure 4C). Further, comparable accumulation in G2/M, indicative of stalled DNA replication forks (Figure 4D), as previously observed for PR-104A [33], suggest a conserved mechanism of action for PR-104A and SN29176 under hypoxia. This conserved hypoxia-selective activity is also observed in vivo, with SN35141 treatment providing greater sterilisation of radiation-resistant hypoxic tumour cells relative to the global tumour cell population (Figure 5C), an effect which was amplified by POR expression. Here, for example, a modest 0.5 log cell kill with single-agent SN35141 was magnified to 2.2 log cell kill post radiation (Figure 5C), with efficacy exceeding that of PR-104 or tirapazamine in the HCT116 sPOR#6 tumour model setting. In a second tumour model, SiHa, the therapeutic activity of SN35141 post radiation was too great to detect any surviving colonies from 5 of 10 tumours (>4 log cell kill), whilst the activity of PR-104 spanning the identified HED range (Figure 1) was evident but remained on scale (2.5–3.7 log cell kill). Collectively, these data indicate that SN35141 is a promising hypoxia-selective prodrug with significant in vivo activity against hypoxic tumour cells. 

Overall, our data suggest that utilising the 2-nitro-4-methylsulfone scaffold avoids human AKR1C3 metabolism and restores the therapeutic ratio of (D)NMBs, thereby restoring the potential for clinical development in the context of hypoxia targeting. However, the common animal models used for pre-clinical toxicology studies (mice, rats and dogs) are unsuitable for testing the safety of SN35141 as they lack functional analogues of human AKR1C3. For example, the closest AKR1C3 orthologues in the mouse, AKR1C6 and AKR1C18, show ~70% amino acid homology to human AKR1C3 (Supplemental Figure S8) and display a divergent pattern of metabolic activities [32]. In contrast, macaque monkey AKR1C3 exhibits 96% amino acid homology to human AKR1C3 [34]. Consistently, this sequence homology translated into functional homology, with macaque AKR1C3 being the only orthologue whose expression in HCT116 cells resulted in increased sensitivity to PR-104A (Figure 7A,B). An immunohistochemical survey of AKR1C3 expression using a commercial macaque normal-tissue microarray (Figure 7C) revealed a staining intensity broadly comparable to human tissues [16]. A caveat of this work is that macaque AKR1C4 was also recognised by the anti-AKR1C3 antibody (Figure 7A), although none of the macaque tissue sections exhibited a staining intensity (H-score) that was atypically greater than the equivalent human tissue, limiting the probability of false positives. This is probably due to the coordinate regulation of AKR1C enzymes by the Nrf2 transcription factor [54]. Our findings indicate that the macaque may represent an appropriate pre-clinical model for guiding the clinical development of SN35141 and related PR-104 analogues such as the clinical candidate CP-506 [40]. Improved tolerability of SN35141, relative to PR-104, in this primate model would indicate that SN35141 could deliver an improved therapeutic ratio in human patients and would therefore represent an attractive HAP candidate for future clinical development.

## 4. Materials and Methods

### 4.1. Test Compounds

PR-104 was supplied by Proacta, Inc., (La Jolla, CA, USA) PR-104A, PR-104H and tetradeuterated derivatives were synthesised, purified and stored as described previously [55,56]. The synthesis of SN29176 and SN35141 is summarised in Scheme 1.

### 4.2. Chemistry Experimental

Elemental analyses were performed by the Campbell Microanalytical Laboratory, University of Otago, Dunedin, New Zealand. Melting points were determined using an Electrothermal IA9100 melting point apparatus and are as read. The ^1^H NMR spectra were measured on a Bruker Avance 400 spectrometer at 400 MHz and were referenced to Me_4_Si or solvent resonances. Chemical shifts and coupling constants were recorded in units of ppm and hertz, respectively. High-resolution electrospray ionisation (HR-ESI-MS) mass spectra were determined on a Bruker micrOTOF-Q II mass spectrometer or an Agilent 6530 Q-TOF mass spectrometer coupled to an Agilent 1200 series HPLC system. Liquid chromatography–mass spectrometry (LCMS) was performed either on an Agilent 1100 LC system interfaced with an Agilent MSD mass detector or on a Micromass Platform LC mass spectrometer. In both cases, mass detection was performed with an APCI source, using simultaneous positive and negative ion acquisition. Column chromatography was performed on silica gel (Merck 230–400 mesh), using the indicated eluents. Thin-layer chromatography was carried out on aluminium-backed silica gel plates (Merck 60 F254), with visualisation of components by UV light (254 nm), with I_2_ or KMnO_4_ staining. Tested compounds (including batches screened in vivo) were 95% pure, as determined by elemental analysis and/or by HPLC conducted on an Agilent 1100 system with diode array detection, using a 150 mm × 3.2 mm Altima 5 mm reversed-phase C8 or C18 column. Elemental analyses indicated by the symbols of the elements were within ± 0.4% of the theoretical values. Compound **1** was first described by Atwell et al. [39] and is commercially available. In the present work it was prepared via a recently reported 3-step procedure [40].

#### Synthesis of SN29176 and SN35141

5-(Bis(2-hydroxyethyl)amino)-4-(methylsulfonyl)-2-nitrobenzoic acid (2). Compound **1** (7.8 g, 29.6 mmol) was dissolved in DMSO (25 mL) and treated with diethanolamine (8.5 mL, 88.8 mmol) at room temperature. The reaction mixture was stirred at room temperature for 2 h then poured into a beaker of ice-cold aqueous HCl (2 M, 100 mL), extracted with EtOAc/i-PrOH (4:1) (3×), washed with brine, dried with Na_2_SO_4_ and concentrated under reduced pressure to give the title compound **2** as a yellow gum (3.6 g, 92%). ^1^H NMR [(CD_3_)_2_SO] δ 14.07 (br s, 1 H), 8.49 (s, 1 H), 7.69 (s, 1 H), 4.61 (br s, 2 H), 3.57–3.54 (m, 4 H), 3.51–3.48 (m, 4 H), 3.46 (s, 3H). HRMS: calculated for C_12_H_17_N_2_O_8_S ([M+H]^+^) 349.0705, found 349.0687.

5-(Bis(2-chloroethyl)amino)-*N*-(2-hydroxyethyl)-4-(methylsulfonyl)-2-nitrobenzamide (3). A stirred solution of compound **2** (490 mg, 1.4 mmol) in SOCl_2_ (12.5 mL) was treated with DMF (3 drops) and heated under reflux for 4 h. The excess SOCl_2_ was removed by distillation under reduced pressure and the residue dissolved in CH_2_Cl_2_ (5 mL) and THF (3 mL), cooled to 0 °C and treated with 2-aminoethanol (296 µL, 4.9 mmol). The reaction mixture was stirred at 0 °C for 20 min then warmed to room temperature, acidified with aqueous HCl (0.5 M, 4 mL) and extracted with EtOAc (2×). The combined organic phases were washed with brine, dried with Na_2_SO_4_ and evaporated to dryness under reduced pressure. The crude product was purified by flash column chromatography on silica gel, eluting with CH_2_Cl_2_/MeOH (25:1). The product obtained was triturated with EtOAc/hexanes to give the title compound **3** as a yellow solid (300 mg, 50%), MP 124–125 °C. ^1^H NMR [(CD_3_)_2_SO] δ 8.80 (t, *J* = 5.7 Hz, 1 H), 8.51 (s, 1 H), 7.69 (s, 1 H), 4.79 (t, *J* = 5.4 Hz, 1 H), 3.81–3.77 (m, 4 H), 3.72–3.69 (m, 4 H), 3.55–3.51 (m, 2 H), 3.48 (s, 3 H), 3.34–3.29 (m, 2 H). APCI MS 429 ([M + H]^+^). C_14_H_19_Cl_2_N_3_O_6_S (calculated): C = 39.26; H = 4.47; N = 9.81; observed: C = 39.45; H = 4.36; N = 9.90.

5-(Bis(2-bromoethyl)amino)-*N*-(2-hydroxyethyl)-4-(methylsulfonyl)-2-nitrobenzamide (SN29176). A solution of compound **3** (250 mg, 0.6 mmol) in 3-methyl-2-butanone (10 mL) was treated with LiBr (1.0 g, 11.8 mmol) and heated to reflux overnight. The reaction mixture was cooled to room temperature and the solvent was removed under reduced pressure. The residue was dissolved in EtOAc and washed with water (3x), dried with Na_2_SO_4_ and concentrated under reduced pressure. The crude mixture was resubmitted to LiBr (2×) and worked up as above. The residue was purified by flash column chromatography on silica gel, eluting with CH_2_Cl_2_/MeOH (20:1). The product obtained was triturated with EtOAc/hexanes to provide the title compound SN29176 as a pale yellow solid (250 mg, 83%), MP 121–123 °C. ^1^H NMR [(CD_3_)_2_SO] δ 8.78 (t, *J* = 5.6 Hz, 1 H), 8.51 (s, 1 H), 7.69 (s, 1 H), 4.79 (t, *J* = 5.4 Hz, 1 H), 3.77–3.74 (m, 4 H), 3.65-3.63 (m, 4 H), 3.56–3.53 (m, 2 H), 3.49 (s, 3 H), 3.34–3.30 (m, 2 H). APCI MS 518 ([M + H]^+^). C_14_H_19_Br_2_N_3_O_6_S.^3^/_10_EtOAc (calculated): C = 33.58; H = 3.97; N = 7.73; observed: C = 33.83; H = 3.78; N = 7.62. Melting point and ^1^H NMR in agreement with values reported in the patent literature [41].

2-(5-(Bis(2-bromoethyl)amino)-4-(methylsulfonyl)-2-nitrobenzamido)ethyl di-*tert*-butyl phosphate (4). To a solution of SN29176 (3.0 g, 5.8 mmol) in DMF (4.1 mL) at 5 °C was added a 1*H*-tetrazole solution (3% in CH_3_CN, 62 mL, 26.7 mmol) followed by di-*tert*-butyl-*N*,*N*-diisopropylphosphoramidite (7.3 mL, 23.2 mmol). The reaction mixture was stirred for 4 h at room temperature, diluted with CH_2_Cl_2_ (25 mL) and cooled to 0 °C before solid *m*-CPBA (70%, 10.2 g, 58.0 mmol) was added portion-wise. The mixture was warmed to room temperature, stirred for a further 1 h, and then the solvents were removed under reduced pressure. The residue was dissolved in EtOAc, washed with a 10% solution of sodium disulfite (2×) then a 5% solution of sodium bicarbonate (3×), dried with Na_2_SO_4_ and concentrated under reduced pressure. The crude product was purified by flash column chromatography on silica gel, eluting with CH_2_Cl_2_/MeOH (25:1) to give the title compound **4** as a yellow gum (2.8 g, 68%). ^1^H NMR [(CD_3_)_2_SO] δ 8.94 (t, *J* = 5.6 Hz, 1 H), 8.53 (s, 1 H), 7.73 (s, 1 H), 4.00–3.96 (m, 2 H), 3.77–3.74 (m, 4 H), 3.64–3.61 (m, 4 H), 3.52–3.48 (m, 2 H), 3.50 (s, 3 H), 1.43 (s, 18 H). HRMS: calculated for C_22_H_36_Br_2_N_3_NaO_9_PS ([M+Na]^+^) 730.0163, found 730.0169.

2-(5-(Bis(2-bromoethyl)amino)-4-(methylsulfonyl)-2-nitrobenzamido)ethyl dihydrogen phosphate (SN35141). Compound **4** (2.7 g, 3.8 mmol) in CH_2_Cl_2_ (14 mL) was cooled to 5 °C and treated with TFA (14 mL). The reaction mixture was stirred for 1 h at room temperature, and the solvent and the excess TFA were removed under reduced pressure. The residue was triturated with CH_2_Cl_2_/iPr_2_O then dissolved in CH_3_CN. The solvent was removed under reduced pressure to provide SN35141 as a yellow gum (2.3 g, 100%). ^1^H NMR [(CD_3_)_2_SO] δ 8.93 (t, *J* = 5.8 Hz, 1 H), 8.52 (s, 1 H), 7.76 (s, 1 H), 3.98–3.93 (m, 2 H), 3.77–3.74 (m, 4 H), 3.64–3.61 (m, 4 H), 3.50–3.45 (m, 2 H), 3.50 (s, 3 H). HRMS: calculated for C_14_H_20_Br_2_N_3_NaO_9_PS ([M+Na]^+^) 617.8899, found 617.8917.

### 4.3. Cell Lines, Cytotoxicity Assays and Multicellular Layer (MCL) Assays 

Cell lines were sourced as summarised in Table S2. STR phenotyping confirmed authenticity. HCT116 cell lines overexpressing AKR1C1-4 [16] and POR [13] had been previously generated and validated for candidate gene expression as described. Cells were maintained in culture under humidified atmospheric conditions with 5% CO_2_ as previously [12], with <3 months cumulative passage from authenticated stocks. Anti-proliferative assays were performed in α-minimal essential medium under aerobic or anoxic conditions, the latter using a 5% H_2_/palladium catalyst scrubbed Bactron anaerobic chamber (Sheldon Manufacturing, Cornelius, OR) to achieve severe anoxia (<10 ppm O_2_ gas phase) during prodrug exposure, as described previously [12]. Total exposure to anoxia did not exceed 6 h, and cells were allowed to regrow for 5 days under drug-free aerobic conditions. MCL assays were performed as described previously [57]. Briefly, MCLs were exposed to 10 µM PR-104A or SN29176 in stirred media compartments under humidified hyperoxic conditions (95% O_2_/5% CO_2_) for 5 h. Cells were dissociated with trypsin, plated at concentrations of 10^2^ to 10^5^ cells per 60 mm dish and grown for 10 days, then colonies were stained with methylene blue. Plating efficiency was calculated relative to drug-free control MCLs.

### 4.4. Clonogenic Survival of Mouse versus Human Bone Marrow Progenitor Cells 

Bone marrow low-density cells harvested from the femori and tibiae of male C57BL/6 mice were isolated on a discontinuous density gradient (1.083 g/mL). Fresh human bone marrow cells (BMCs) were sourced from Lonza Bioscience (Houston, TX, USA). Primary BM progenitors were exposed to PR-104A (50 μM, 3.5 h) under aerobic conditions (37 °C, 5% CO_2_), washed and plated in MethoCult H4434 (human) or MethoCult M3434 (mouse) (Stem Cell Technologies, Cambridge, MA, USA) for two weeks’ growth with subsequent colony counting.

### 4.5. Prodrug Metabolism by Recombinant AKR1C3 

Recombinant AKR1C3 was diluted to 2 μmol/L in potassium phosphate buffer (100 mmol/L (pH 7.4), 37 °C) containing NADPH (100 μmol/L), bovine serum albumin (0.5 mg/mL) and either PR-104A or SN29176 (50 μmol/L). Reactions (400 μL) were monitored by absorbance (340 nm) using an Agilent 8453E diode array spectrophotometer to measure NADPH consumption. Rates of absorbance change over 5 min were determined. For heat inactivation, recombinant AKR1C3 was denatured at 95 °C for 5 min. 

Steady-state enzyme kinetics with recombinant AKR1C3 were measured by monitoring reduction at a wavelength of 400 nm, with kinetic parameters calculated for each (D)NBM using the following extinction coefficients: PR-104A, 6000 M^−1^ cm^−1^; SN27686 (3,5-DNBM, X = Br; Figure S1), 7000 M^−1^ cm^−1^; SN28099 (2,4-DNBM, X = OMs; Figure S1), 4000 M^−1^ cm^−1^. Reactions were performed in 60 μL in UVettes™ (Eppendorf, Hamburg, Germany) containing 10 mM Tris–Cl (pH 7.0), 4% DMSO, 0.25 mM NAD(P)H and varying substrate concentrations. Reactions were commenced by addition of enzyme and substrate concentrations that were varied from ~0.2× to 5× *K*_M_, based on initial empirical observations. Nonlinear regression analyses and Michaelis–Menten curve fitting were performed using SigmaPlot™ 10.0 (Systat Software, Richmond, CA, USA).

### 4.6. Candidate Gene Expression

Synthetic cDNAs were commercially sourced as indicated in Table S3 and cloned into the vector F279-V5, which shares an identical backbone with F527-V5 described previously [16] but utilises the cytomegalovirus (CMV) early immediate promoter. HCT116 cells were transfected using Fugene6 and pooled stable populations selected with puromycin, as previously described [58]. Protein expression was confirmed through induction of an occult COOH-terminal V5-tag (Tag-on-Demand, Invitrogen), as described previously [16]. Functional AKR1C3 activity was established by incubating cells with the fluorogenic AKR1C probe coumberone (5 μmol/L, 37 °C, 3 h) [59] using a fluorescent plate reader (Molecular Devices SpectraMax M2; Ex/Em 385/510 nm) and standard curves of authentic reduced coumberone. Functional POR expression was established through cytochrome c reduction assays, as described [60].

### 4.7. Flow Cytometry

For detection of γH2AX, HCT116 wild-type (WT) and HCT116 POR cells were exposed to PR-104A or SN29176 for 4 h under aerobic or anoxic conditions. Diphenyliodonium (DPI)-treated samples were exposed to 100 µmol/L DPI 2 h before drug exposure. After drug incubation, cells were washed free of the drug and incubated for 20 h under aerobic conditions. Cells were fixed in 4% paraformaldehyde, permeabilised in 0.2% Triton X-100/1% BSA (in PBS) and exposed to an AF647-conjugated phospho-Ser139 H2AX (γH2AX) antibody (Becton Dickinson). Cells were analysed by flow cytometry using a BD LSR II and corresponding BD FACSDiva software. Fluorescence intensity of propidium iodide (PI) was detected in C33A cells after incubation with PR-104A or SN29176 for 4 h under anoxic conditions, followed by 20 h post-treatment recovery under aerobic conditions. Cells were analysed by flow cytometry using a BD LSR II and corresponding BD FACSDiva software.

### 4.8. Western Immunoblotting

Cell lysates were prepared in radioimmunoprecipitation assay buffer, and 10 µg of protein was loaded on SDS-PAGE gel, transferred, blocked and probed with primary monoclonal antibodies for AKR1C3 (1:5000 dilution clone NP6.G6.A6; Sigma Aldrich, St. Louis, MO, USA) or V5 tag epitope (1:5000 dilution, Invitrogen, New Zealand) and detected using chemiluminescent ECL detection (Supersignal, Thermo Scientific, Auckland, New Zealand).

### 4.9. Animals, Excision and Growth Delay Assays

Specific pathogen-free female NIH-III (NIH-Lyst^bg^ Foxn1^nu^ Btk^xid^) nude mice were obtained from Charles River Laboratories (Wilmington, MA, USA), bred in the Vernon Jansen Unit (shared vivarium, University of Auckland), and supplied at 7–9 weeks of age. Animal protocols were approved by the University of Auckland Animal Ethics Committee (CR830 and CR1190). Animal handling and determination of the maximum tolerated dose of drug were as described previously [12]. Tumours were grown in the flanks of female NIH-III nude mice by subcutaneous injection of 10^7^ cells with random treatment group assignment. For excision assays, upon reaching a tumour size of ~400 mm^3^, mice were injected with a single intraperitoneal dose of pre-prodrug or vehicle. Treatment and sample collection were as described previously [12]. Statistical analysis was undertaken using the Holm–Sidak one way ANOVA (SigmaStat v3.0). For growth delay assays, mice received a single intraperitoneal dose of pre-prodrug once tumours reached ~200 mm^3^. Growth delays were conducted as described previously [12]. The median time for tumours to increase in volume 4-fold relative to pre-treatment volume was determined (RTV-4), and the specific growth delay was calculated as the percentage increase in RTV-4 for treated versus control groups. Statistical analysis was performed using Dunn’s one way ANOVA (SigmaStat v3.0).

### 4.10. Immunostaining 

Xenografts were established in NIH-III nude mice; formalin-fixed tumours were sectioned (5 μm) and immunostained using anti-AKR1C3 (Sigma) or anti-POR (Santa Cruz Biotech, Dallas, Texas, USA) monoclonal antibodies as described previously [15,16]. Primary antibodies were visualised with an EnVision Dual Link HRP/DAB Kit (Dako). For immunofluorescence detection of hypoxia, mice were dosed i.p. with 60 mg/kg pimonidazole (Hypoxyprobe-1 Kit; Hypoxyprobe Inc, Burlington, MA, USA) 90 min prior to removal of tumours, and adducts were detected using the associated fluorescent pimonidazole antibody, as described previously [16]. Immunofluorescence detection of AKR1C3 was performed using an anti-AKR1C3 antibody conjugated to Alexa Fluor 488. Immunostaining of the macaque normal tissue array (ASM221, Pantomics Inc., Fairfield, California, USA) for AKR1C3 was performed as described previously [16]. Cores were scored for staining intensity and proportion of AKR1C3-positive neoplastic cells by a certified pathologist (NKL) using a semi-quantitative measure on a 4-point scale ranging from negative (score 0) to strong staining (score 3). This measure was applied to the epithelial elements within the normal TMA. Histochemical scores (H-scores) for AKR1C3 were determined using the following equation: (percentage of intensity 1 cells × 1) + (percentage of intensity 2 cells × 2) + (percentage of intensity 3 cells × 3) = H-score (maximum = 300). 

## 5. Conclusions

Structure-based design of SN35141, based on the clinical stage compound PR-104 yielded a novel prodrug with selective cytotoxicity for hypoxic tumour cells in vitro and in vivo. Collectively, data demonstrated that avoidance of aerobic metabolism by human AKR1C3 is likely to be sufficient to prevent the myelotoxicity observed clinically with PR-104. Human and macaque AKR1C3 were able to bioactivate PR-104A but not SN29176, reflecting the high sequence and functional homology of the AKR1C family members between human and macaque [34]. The conserved haemopoietic system of these primate species [61] indicates that it may be possible to identify models that will more accurately reflect the haematological toxicity observed with AKR1C3-activated prodrugs such as PR-104. 

## 6. Patents

Patents arising from this work include the international PCT applications WO2005042471A1 and WO2014031012A1. The latter was assigned to Health Innovation Ventures, leading to the issued patents EP2888227B1, US10202408B2, CA2886574C, US9873710B2, AU2013/306514B2 and US9505791B2.

## Data Availability

Data is contained within the article and Supplementary Material.

## Supplementary Materials

The following are available online at https://www.mdpi.com/article/10.3390/ph14121231/s1: Supplementary Table S1. Relationship between PR-104 input dose and PR-104A plasma exposure (calculated AUC_free_) in mouse or human subjects; Supplementary Table S2. Source of cell lines; Supplementary Table S3. Source of cDNAs sequences for expression studies; Supplementary Figure S1. Recombinant human AKR1C3 assay of three prodrug isomer classes: the 2,4-dinitro, the 3,5-dinitro and the 2,6-dinitro benzamide mustards (DNBMs); Supplementary Figure S2. Flow cytometry detection of γH2AX following aerobic incubation of HCT116 WT and HCT116 POR cells to prodrugs in the presence or absence of 100 µM DPI; Supplementary Figure S3. Flow cytometry detection of γH2AX following aerobic incubation of HCT116 WT and HCT116 POR cells to prodrugs in the presence or absence of 100 µM DPI; Supplementary Figure S4. Selection of HCT116 POR clones for in vivo studies; Supplementary Figure S5. Ex vivo evaluation of HCT116 POR clones; Supplementary Figure S6. Selection of HCT116 AKR1C3 clones for in vivo studies; Supplementary Figure S7. Comparative in vivo metabolism of PR-104 by HCT116 WT, sPOR#6 and AKR1C3#6 xenografts; Supplementary Figure S8. Sequence alignment of AKR1C orthologues from different species.
