# Supplementary material for: Restoring Tumour Selectivity of the Bioreductive Prodrug PR-104 by Developing an Analogue Resistant to Aerobic Metabolism by Human Aldo-Keto Reductase 1C3
Source: Pharmaceuticals (Basel). 2021 Nov 26;14(12):1231. doi: 10.3390/ph14121231 (PMC8707548; doi:10.3390/ph14121231)
Supplement: Supplementary file 1 [file pharmaceuticals-14-01231-s001.zip › pharmaceuticals-1467045-supplementary.pdf]

pharmaceuticals

Supplementary data

Restoring tumour selectivity of the prodrug PR-104 through development of an analogue resistant to aerobic metabolism by human aldo-keto reductase 1C3

Maria R Abbattista, Amir Ashoorzadeh, Christopher P Guise, Alexandra M Mowday, Rituparna Mittra, Shevan Silva, Kevin O Hicks, Matthew R Bull, Victoria Jackson-Patel, Xiaojing Lin, Gareth A Prosser, Neil K Lambie, Gabi U Dachs, David F Ackerley, Jeff B Smaill and Adam V Patterson

Contents:

Supplementary Table S1. Relationship between PR-104 input dose and PR-104A plasma exposure (calculated AUC_{free}) in mouse or human subjects.

Supplementary Table S2. Source of cell lines

Supplementary Table S3. Source of cDNAs sequences for expression studies

Supplementary Figure S1. Recombinant human AKR1C3 assay of three prodrug isomer classes; the 2,4-dinitro, the 3,5-dinitro and the 2,6-dinitro benzamide mustards (DNBMs).

Supplementary Figure S2a. IC50 values demonstrating inhibition of AKR1C3 by SN34037 did not alter sensitivity to SN29176, while it prevented sensitivity to PR-104A in a manner directly proportional to endogenous AKR1C3 expression.

Supplementary Figure S2b. Loss of clonogenic viability in HCT116 POR cells following exposure to PR-104A or SN29176 under anoxic conditions, with or without diphenyl iodonium (DPI, 100 μM) pre-treatment.

Supplementary Figure S3. Flow cytometry detection of γH2AX following aerobic incubation of HCT116 WT and HCT116 POR cells to prodrugs in the presence or absence of 100 μM DPI.

Supplementary Figure S4. Selection of HCT116 POR clones for *in vivo* studies.

Supplementary Figure S5. *Ex-vivo* evaluation of HCT116 POR clones

Supplementary Figure S6. Selection of HCT116 AKR1C3 clones for *in vivo* studies.

Supplementary Figure S7. Comparative *in vivo* metabolism of PR-104 by HCT116 WT, sPOR#6 and AKR1C3#6 xenografts.

Supplementary Figure S8. Sequence alignment of AKR1C orthologues from different species.

Supplementary Table S1. Relationship between PR-104 input dose and PR-104A plasma exposure (calculated AUC_{free}) in mouse or human subjects.

Mouse i.p. (dose)		Mouse PR-104A (predicted AUC_{free})*	Human i.v. 1 h infusion (dose)	Human PR-104A (predicted AUC_{free})#
μmol/kg	mg/kg	μM.h	mg/m²	μM.h
0.56	0.33	0.03	1.30	0.03
1.43	0.83	0.05	2.23	0.05
1.95	1.13	0.06	2.95	0.06
2.66	1.54	0.08	3.95	0.09
3.63	2.10	0.11	5.32	0.12
4.24	2.46	0.13	6.20	0.14
4.96	2.87	0.15	7.23	0.16
5.79	3.35	0.18	8.45	0.19
6.76	3.92	0.21	9.91	0.22
14.71	8.52	0.47	22.53	0.49
23.44	13.57	0.79	37.79	0.83
27.38	15.85	0.95	45.08	0.99
37.35	21.63	1.36	64.54	1.41
43.63	25.26	1.63	77.45	1.70
50.97	29.51	1.96	93.14	2.04
59.53	34.47	2.36	112.23	2.46
69.54	40.26	2.85	135.00	2.96
123.49	71.50	5.10	215.52	4.72
189.98	110.00	8.52	354.00	7.75
211.14	122.25	10.06	455.56	9.98
241.80	140.00	12.38	550.00	12.05
281.52	163.00	15.52	770.00	16.86
351.90	203.75	21.47	962.94	21.09
381.69	221.00	24.14	1100.00	24.09
422.28	244.50	27.92	1400.00	30.66
492.66	285.25	34.86	1659.13	36.33
563.04	326.00	42.30	2013.15	44.09
619.34	358.60	48.60	2313.34	50.66
675.65	391.20	55.23	2628.62	57.57
731.95	423.80	62.17	2958.99	64.80
844.56	489.00	77.00	3665.00	80.26
1013.47	586.80	101.63	4837.16	105.93
1126.08	652.00	119.63	5694.05	124.70
1182.38	684.60	129.11	6145.13	134.58
1238.69	717.20	138.91	6611.29	144.79
1294.99	749.80	149.02	7092.55	155.33
1329.88	770.00	155.44	7398.32	162.02

1462.87	847.00	181.05	8617.01	188.71
1595.85	924.00	208.42	9919.89	217.25
1994.82	1155.00	301.15	14333.56	313.90
2260.79	1309.00	371.82	17696.89	387.56

* Free fraction in mouse plasma = 69.9 ± 3.1 % [mean ± sem]
# Free fraction in human plasma = 43.1 ± 5.0 % [mean ± sem]

Supplementary Table S2. Sources of cell lines

**Cell line**	**Tumour type**	**Supplier**
C33A	Cervix	Onyx Pharmaceuticals, CA
H1299	NSCLC	ATCC
H460	NSCLC	ATCC
HCT116	Colon	ATCC
SiHa	Cervix	ATCC
MDA-468	Breast	ATCC

Supplementary Table S3. Source of cDNAs sequences for expression studies

**Gene**	**Source**	**Reference sequence**
POR	Invitrogen Ultimate ORF (IOH21456)	NCBI ref seq BC034277.1
Mouse AKR1C6	Genscript (human codon optimised)	NCBI ref seq NM_030611.3
Mouse AKR1C18	Genscript (human codon optimised)	Genbank ref seq AB059565.1
Rat AKR1C14	Genscript (human codon optimised)	NCBI ref seq NM_138547.2
Rat AKR1C18	Genscript (human codon optimised)	NCBI ref seq NM138510.1
Dog AKR1C3	Genscript (human codon optimised)	NCBI ref seq NM001012344.1
Macaque AKR1C1	Genscript (human codon optimised) - Corresponds to *macaca mulatta* (rhesus)	NCBI ref seq NM_001195574.1

Macaque AKR1C3	Genscript (human codon optimised) - Corresponds to *macaca mulatta* (rhesus)	NCBI ref seq XM_001104543.2
Macaque AKR1C4	Genscript (human codon optimised) - Corresponds to *macaca mulatta* (rhesus)	NCBI ref seq NM_001195575.1
Human AKR1C1	Invitrogen Ultimate ORF (IOH14162)	NCBI ref seq NM_001353.5
Human AKR1C2	Invitrogen Ultimate ORF (IOH57036)	NCBI ref seq NM_001354.5
Human AKR1C3	Invitrogen Ultimate ORF (IOH13880)	NCBI ref seq NM_003739.5
Human AKR1C4	Origene (SC319841)	NCBI ref seq NM_001818.3

Supplementary Figure S1. Recombinant human AKR1C3 assay of three prodrug isomer classes; the 2,4-dinitro, the 3,5-dinitro and the 2,6-dinitro benzamide mustards (DNBMs).

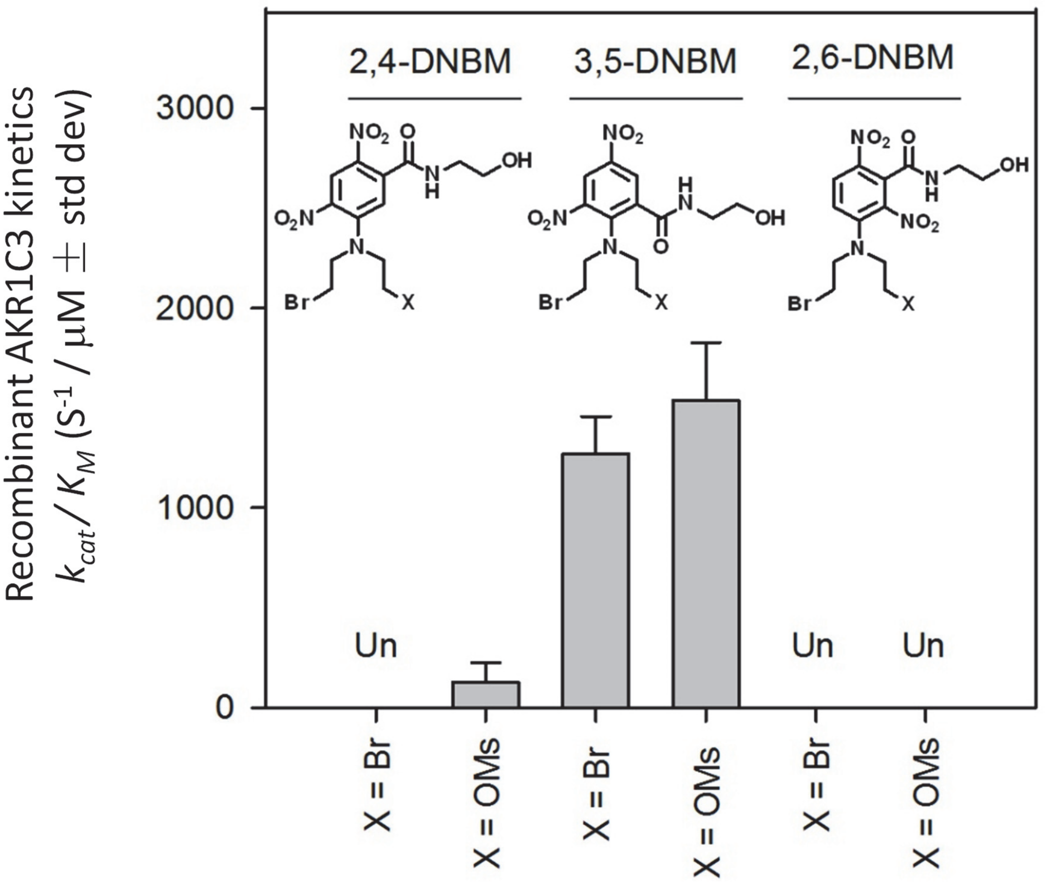

Supplementary Figure S2a. IC50 values demonstrating inhibition of AKR1C3 by SN34037 did not alter sensitivity to SN29176, while it prevented sensitivity to PR-104A in a manner directly proportional to endogenous AKR1C3 expression.

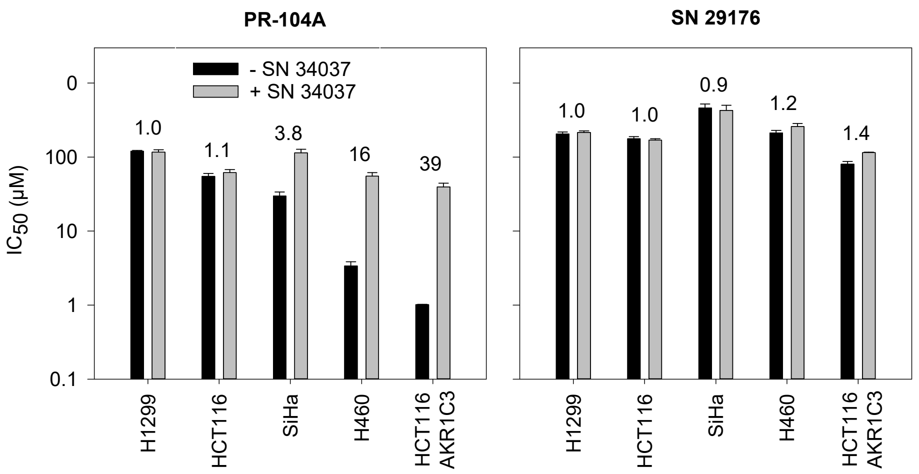

Supplementary Figure S2b. Loss of clonogenic viability in HCT116 POR cells following exposure to PR-104A or SN29176 under anoxic conditions, with or without diphenyl iodonium (DPI, 100 μM) pre-treatment.

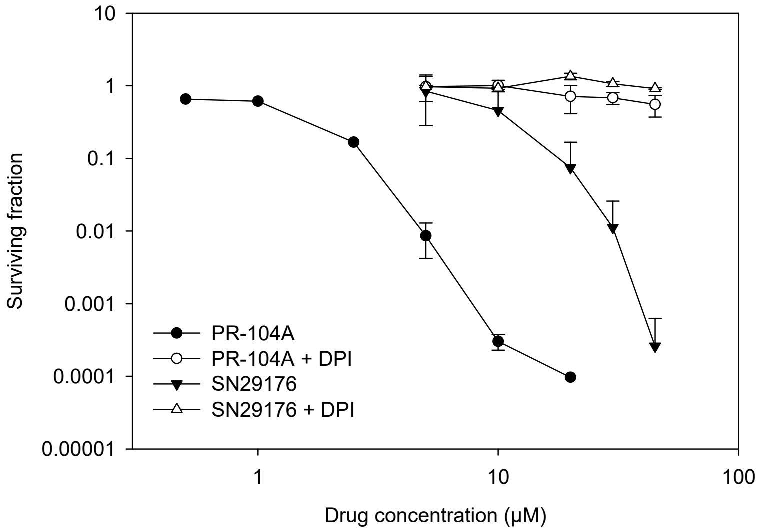

Supplementary Figure S3. Flow cytometry detection of γH2AX following aerobic incubation of HCT116 WT and HCT116 POR cells to prodrugs in the presence or absence of 100 μM DPI.

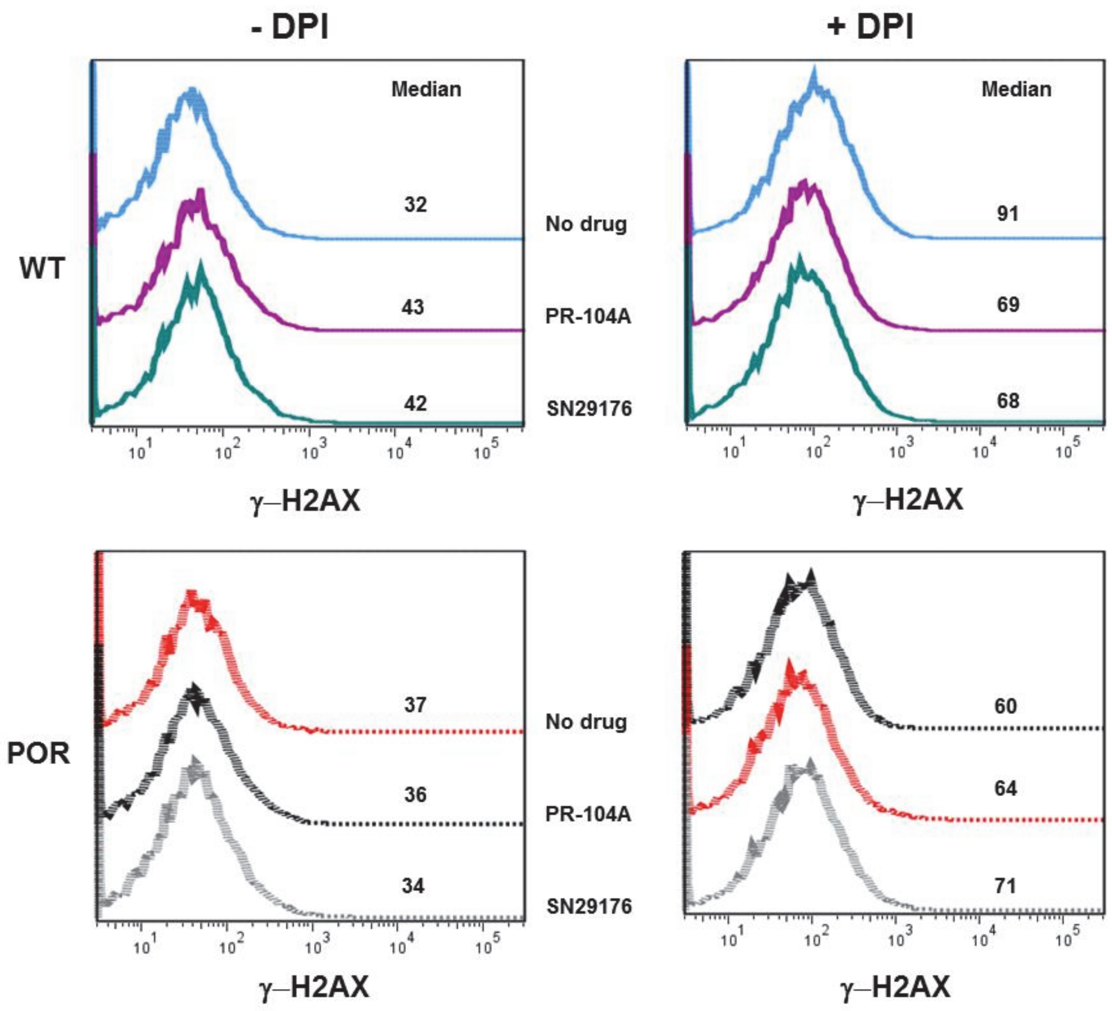

Supplementary Figure S4. Selection of HCT116 POR clones for *in vivo* studies.

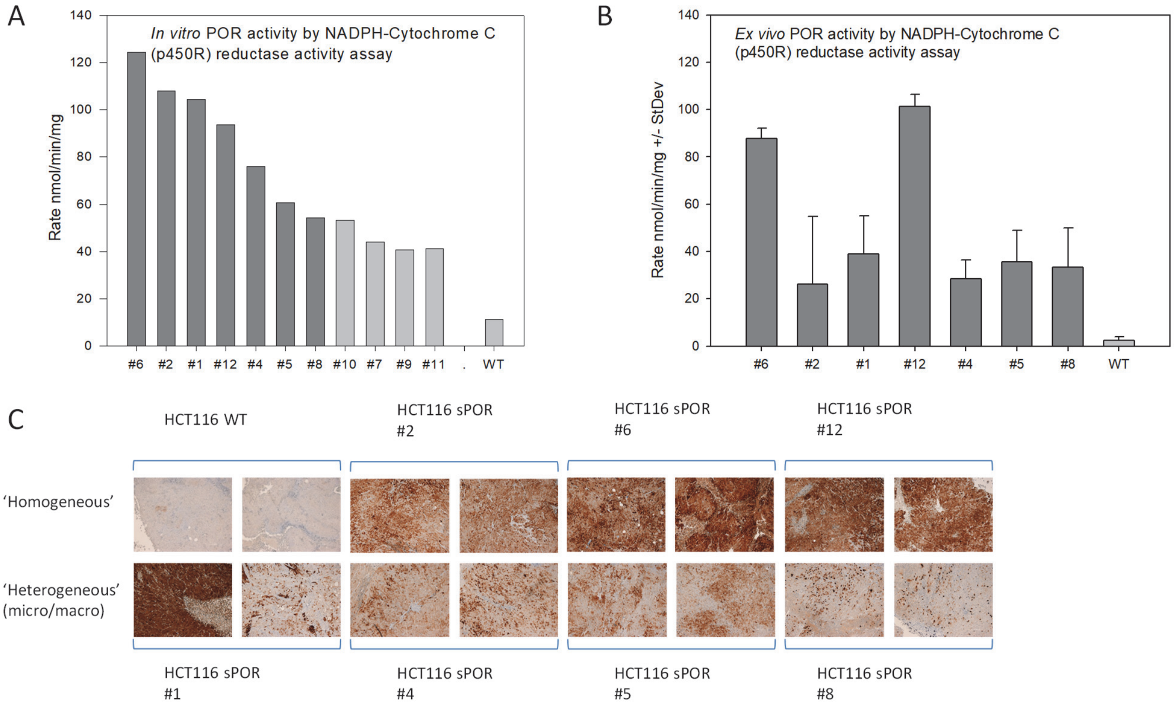

Supplementary Figure S5. *Ex-vivo* evaluation of HCT116 POR clones

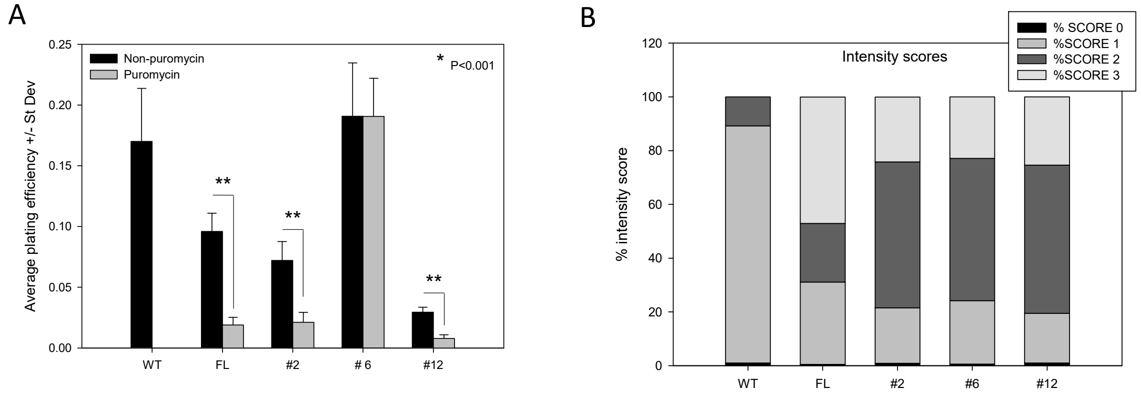

A) Comparative *ex-vivo* plating efficiency of clonal POR tumour cells with or without puromycin (3 μM) selection. B) Percentage of cancer positive stained cells in three fields per sample was measured. Staining intensity was scored as 1 (weak), 2 (moderate) and 3 (strong). Moderate staining intensity showed the highest frequency in all clones.

Supplementary Figure S6. Selection of HCT116 AKR1C3 clones for *in vivo* studies.

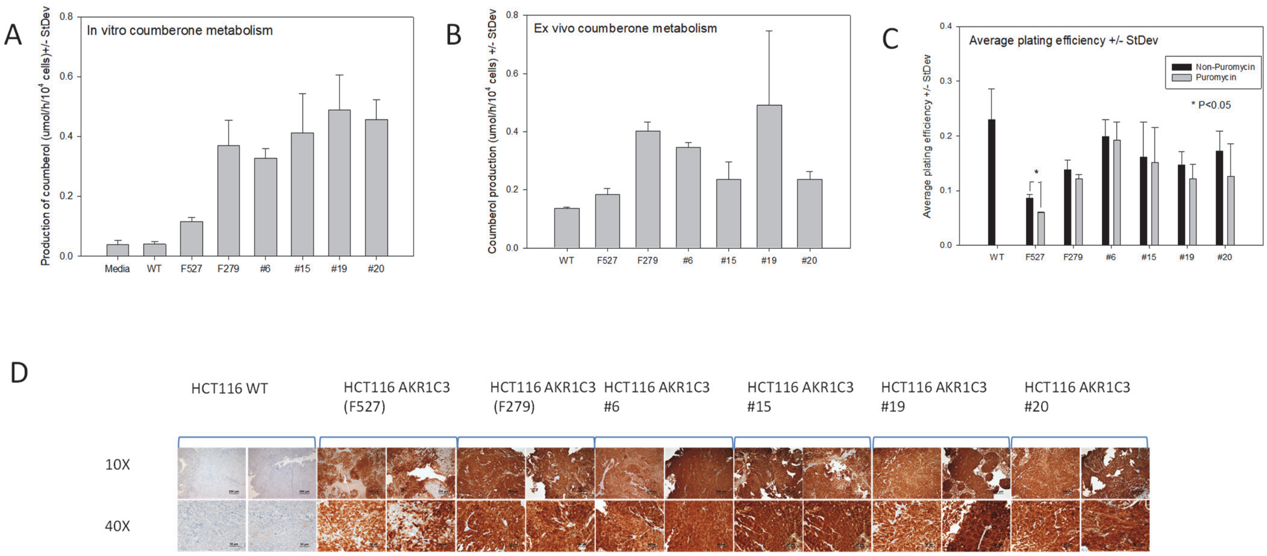

Plating efficiency was calculated in non-selective vs selective media to determine the proportion of cells that retain the puromycin selection marker expressed from the F279 V5 plasmid.

Supplementary Figure S7. Comparative *in vivo* metabolism of PR-104 by HCT116 WT, sPOR#6 and AKR1C3#6 xenografts.

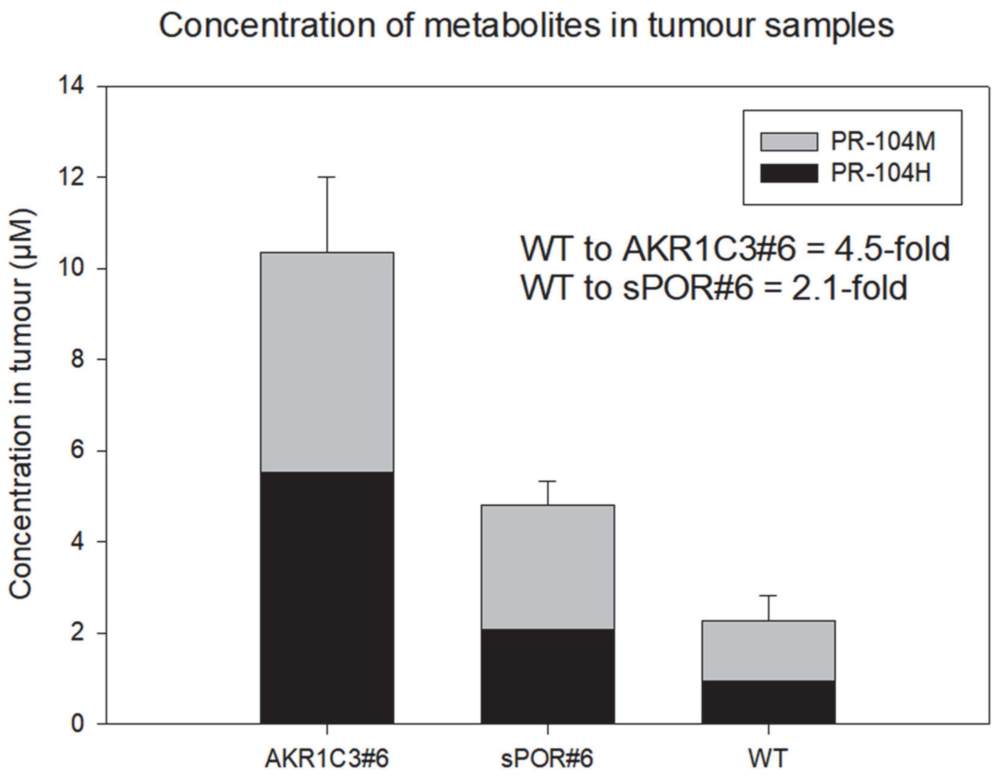

Supplementary Figure S8. Sequence alignment of AKR1C orthologues from different species.

Percentage amino acid homologies relative to human AKR1C3 are 96 % (macaque AKR1C3), 87 % (macaque AKR1C1), 86 % (macaque AKR1C4), 68 % (dog AKR1C3), 71 % (rat AKR1C18), 70 % (rat AKR1C14/rat AKR1C9), 73 % (mouse AKR1C18) and 76 % (mouse AKR1C6). Macaque sequences are from the Rhesus Macaque (*Macaca mulatta*) although the amino acid sequence of AKR1C3 from the Cynomolgus Macaque (*Macaca fascicularis*) are identical.

```
Hum 1C3   MDS-KHQCVKLNDGHFMPVLGFGTYAPPEVPRSKALEVTKLAIEAGFRHIDSAHLYNNEEQVGLAI
Mac 1C3   MDS-KHQRVKLNDGHFMPVLGFGTYAPPEVPRSKALEVTKLAIEAGFRHIDSAHLYNNEEQVGLAI
Mac 1C1   MDS-KHQCVKLNDGHFMPVLGFGTYAPAEVPKNKAIEATKLAIEAGFRHIDSAHLYNNEEYVGLAI
Mac 1C4   MDS-KHQRVKLNDGHFMPVLGFGTYAPVEVPKDKALEATKLAIEVGFRHVDCAYAYNNEEYVGLAI
Dog 1C3   MNLMKLRSVKLNDGLSMPPLGFGTSAPSKVPKTEVEEAVKRAIDVGYRHFDSAYMYLNEEEIGRAI
Rat 1C18  MNS-KIQKMELNDGHSIPVLGFGTYATEENLRKKSMESTKIAIDVGFRHIDCSHLYQNEEEIGQAI
Rat 1C14  MDS-ISLRVALNDGNFIPVLGFGTTVPEKVAKDEVIKATKIAIDNGFRHFDSAYLYEVEEEVGQAI
Mou 1C18  MNS-KIQKIELNDGHSIPVLGFGTYATEEHLKKKSMESTKIAIDVGFCHIDCSHLYQNEEEIGQAI
Mou 1C6   MDS-KQQTVRLSDGHFIPILGFGTYAPQEVPKSKATEATKIAIDAGFRHIDSASMYQNEKEVGLAI

Hum 1C3   RSKIADGSVKREDIFYTSKLWSTFHRPELVRPALENSLKKAQLDYVDLYLIHSPMSLKPGEELSPT
Mac 1C3   RSKIADGTVKREDIFYTSKLWSTFHRPELVRPALENSLKKAQLDYVDLYLIHSPVSLKPGEELSPT
Mac 1C1   RSKIADGTVKREDIFYTSKLWCNSHRPEFVRPALERSLKNLQLDYVDLYLIHFPVSLKPGEELIPK
Mac 1C4   RSKIADGTVKREDIFYTSKLWCNSHRPELVRPALERSLKNLQLDYVDLYLIHSPVSLKPGEELIPK
Dog 1C3   QRKIADGTVKREDIFYTSKVWVTFLRPELVQTNLEMSLKKLGFSYVDLYLIHFPVPLKPGEELFPK
Rat 1C18  VSKIEDGTVKREDIFYTSKLWSTSHRPELVRPSLENSLRKLNLDYVDLYLIHFPVSLKPGDELLPQ
Rat 1C14  RSKIEDGTVKREDIFYTSKLWSTFHRPELVRTCLEKTLKSTQLDYVDLYIIHFPMALQPGDIFFPR
Mou 1C18  LSKIEDGTVKREDIFYTSKLWSTSHRPELVRPSLENSLRKLNLDYVDLYLIHFPVSLKPGNELLPK
Mou 1C6   RSKIADGTVKREDIFYTSKVWCTFHRPELVRVCLEQSLKQLQLDYVDLYLIHFPMAMKPGENYLPK

Hum 1C3   DENGKVIFDIVDLCTTWEAMEKCKDAGLAKSIGVSNFNRRQLEMILNKPGLKYKPVCNQVECHPYF
Mac 1C3   DENGKLIFDIVDLCTTWEAMEKCKDAGLAKSIGVSNFNHRQLEMILNKPGLKYKPVCNQVECHPYF
Mac 1C1   DENGKLLFDTVDLCATWEAMEKCKDAGLAKSIGVSNFNRRQLEMILNKPGLKYKPVCNQVECHPYL
Mac 1C4   DENGKVLFDTVDLCATWEAMEKCKDAGLAKSIGVSNFNRRQLEMILNKPGLKYKPVCNQVECHPYF
Dog 1C3   DKDGKIIFDRVDLCATWEAMEKCKDSGLAKSIGVSNFNRRQLERILSKPRLKYKPVCNQVECHLYF
Rat 1C18  DEHGNLILDTVDLCDTWEAMEKCKDAGLAKSIGVSNFNRRQLEKILNKPGLKHRPVCNQVECHLYL
Rat 1C14  DEHGKLLFETVDICDTWEAMEKCKDAGLAKSIGVSNFNCRQLERILNKPGLKYKPVCNQVECHLYL
Mou 1C18  DEHGNLIFDTVDLCDTWEAMEKCKDAGLAKSIGVSNFNRRQLEMILNKPGLKYKPVCNQVECHLYL
Mou 1C6   DENGKLIYDAVDICDTWEAMEKCKDAGLAKSIGVSNFNRRQLEKILKKPGLKYKPVCNQVECHPYL

Hum 1C3   NRSKLLDFCKSKDIVLVAYSALGSQRDKRWVDPNSPVLLEDPVLCALAKKHKRTPALIALRYQLQR
Mac 1C3   NQSKLLDFCKSKDIVLVAYSALGSQRDKRWVDQNSPVLLEDPVLCALAKKHKRTPALIALRYQLQR
Mac 1C1   NQRKLLDFCKSKDIVLVAYSALGSHREKPWVDQNSPVLLEDPVLCALAKKHKRTPALIALRYQLQR
Mac 1C4   NQRKLLDFCKSKDIVLVAFSALGSHREKQWVDQNSPVLLEDPVLCALAKKHKQTPALIALRYQLQR
Dog 1C3   NQSKLLEFCKSKDIILTAYGALGSDFGKEWVNQDAPVLLKDPVLNAVAARHGRTPAQVALRFQLQR
Rat 1C18  NQSKLLAYCKMNDIVLVAYGALGTQRYKYCINEDTPVLLDDPILCTMAKKYKRTPALIALRYQLER
Rat 1C14  NQSKMLDYCKSKDIILVSYCTLGSSRDKTWVDQKSPVLLDDPVLCAIAKKYKQTPALVALRYQLQR
Mou 1C18  NQSKLLAYCKMNDIVLVAYGALGTQRYKYCINEDTPVLLDDPVLCAMAKKYKRTPALIALRYQLDR
Mou 1C6   NQGKLLDFCRSKDIVLVAYSALGSHREKQWVDQSSPVLLDNPVLGSMAKKYNRTPALIALRYQLQR

Hum 1C3   GVVVLAKSYNEQRIRQNVQVFEFQLTAEDMKAIDGLDRNLHYFNSDSFASHPNYPYSDEY
Mac 1C3   GVVVLAKSYNEQRIRENVQVFEFQLTSEDMKAIDGLNRNLRYFNSDSLASHPNYPYSDEY
Mac 1C1   GVVVLAKSYNEQRIRENMKVFEFQLTSEDMKAIDGLDRNIRYLTLDIFAGPPNYPFSDEY
Mac 1C4   GVVVLAKSYTEQRIRENMKVFEFQLTSEDMKAIDGLDRNIRYLTLDILADSPNYPYSDEY
Dog 1C3   GVVALAKSFNEKRIRENFQVFDFQLTPEDMETLSSLNKNIRYFSDTLFATHPDYPFNDED
Rat 1C18  GIVTLVKSFNEERIRENLQVFDFQLASDDMEILDNLDRNLRYFPANMFKAHPNFPFSDEY
Rat 1C14  GVVPLIRSFNAKRIKELTQVFEFQLASEDMKALDGLNRNFRYNNAKYFDDHPNHPFTDE
Mou 1C18  GIVALAKSFNEERIRENMQVFDFQLASDDMKILDGLDRNLRYFPADMFKAHPNFPFFDEY
Mou 1C6   GVVVLAKSFSEKRIKENMQVFEFQLTSEDMKVLDDLNKNIRYISGSSFKDHPDFPFWDEY
```
